# Phage defence loci of *Streptococcus thermophilus—*tip of the anti-phage iceberg?

**DOI:** 10.1093/nar/gkae814

**Published:** 2024-09-24

**Authors:** Philip Kelleher, Guillermo Ortiz Charneco, Zoe Kampff, Natalia Diaz-Garrido, Francesca Bottacini, Brian McDonnell, Gabriele A Lugli, Marco Ventura, Alexey Fomenkov, Pascal Quénée, Saulius Kulakauskas, Paul de Waal, Noël N M E van Peij, Christian Cambillau, Richard John Roberts, Douwe van Sinderen, Jennifer Mahony

**Affiliations:** School of Microbiology and APC Microbiome Ireland, University College Cork, Cork T12 YT20, Ireland; School of Microbiology and APC Microbiome Ireland, University College Cork, Cork T12 YT20, Ireland; School of Microbiology and APC Microbiome Ireland, University College Cork, Cork T12 YT20, Ireland; School of Microbiology and APC Microbiome Ireland, University College Cork, Cork T12 YT20, Ireland; Department of Biological Sciences, Munster Technological University, Cork, Ireland; School of Microbiology and APC Microbiome Ireland, University College Cork, Cork T12 YT20, Ireland; Laboratory of Probiogenomics, Department of Chemistry, Life Sciences and Environmental Sustainability, and Interdepartmental Research Centre Microbiome Research Hub, University of Parma, Parma, Italy; Laboratory of Probiogenomics, Department of Chemistry, Life Sciences and Environmental Sustainability, and Interdepartmental Research Centre Microbiome Research Hub, University of Parma, Parma, Italy; New England Biolabs, Ipswich, MA, USA; Université Paris-Saclay, INRAE, AgroParisTech, Micalis Institute, Jouy-en-Josas, France; Université Paris-Saclay, INRAE, AgroParisTech, Micalis Institute, Jouy-en-Josas, France; DSM-Firmenich, Taste, Texture & Health, Center for Food Innovation, Alexander Fleminglaan 1, 2613 AX Delft, The Netherlands; DSM-Firmenich, Taste, Texture & Health, Center for Food Innovation, Alexander Fleminglaan 1, 2613 AX Delft, The Netherlands; School of Microbiology and APC Microbiome Ireland, University College Cork, Cork T12 YT20, Ireland; Laboratoire d’Ingénierie des Systèmes Macromoléculaires (LISM), Institut de Microbiologie, Bioénergies et Biotechnologie (IMM), Aix-Marseille Université – CNRS, UMR 7255, Marseille, France; New England Biolabs, Ipswich, MA, USA; School of Microbiology and APC Microbiome Ireland, University College Cork, Cork T12 YT20, Ireland; School of Microbiology and APC Microbiome Ireland, University College Cork, Cork T12 YT20, Ireland

## Abstract

Bacteria possess (bacterio)phage defence systems to ensure their survival. The thermophilic lactic acid bacterium, *Streptococcus thermophilus*, which is used in dairy fermentations, harbours multiple CRISPR-Cas and restriction and modification (R/M) systems to protect itself against phage attack, with limited reports on other types of phage-resistance. Here, we describe the systematic identification and functional analysis of the phage resistome of *S. thermophilus* using a collection of 27 strains as representatives of the species. In addition to CRISPR-Cas and R/M systems, we uncover nine distinct phage-resistance systems including homologues of Kiwa, Gabija, Dodola, defence-associated sirtuins and classical lactococcal/streptococcal abortive infection systems. The genes encoding several of these newly identified *S. thermophilus* antiphage systems are located in proximity to the genetic determinants of CRISPR-Cas systems thus constituting apparent Phage Defence Islands. Other phage-resistance systems whose encoding genes are not co-located with genes specifying CRISPR-Cas systems may represent anchors to identify additional Defence Islands harbouring, as yet, uncharacterised phage defence systems. We estimate that up to 2.5% of the genetic material of the analysed strains is dedicated to phage defence, highlighting that phage-host antagonism plays an important role in driving the evolution and shaping the composition of dairy streptococcal genomes.

## Graphical Abstract

**Graphical Abstract:**
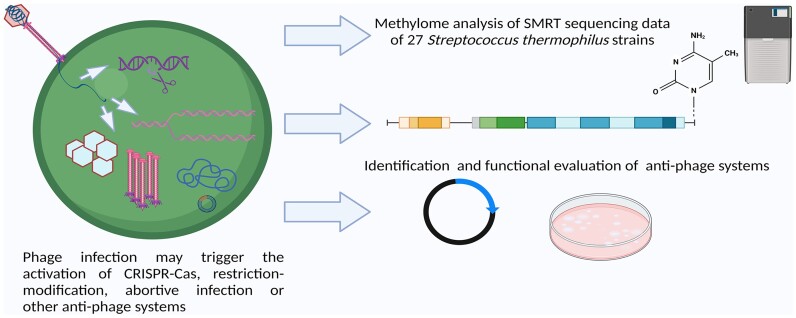

## Introduction

*Streptococcus thermophilus*, a member of the viridans group of streptococci, is widely exploited in the dairy industry owing to its associated technological properties (1). The intensive application of strains of this species and continuous cultivation under optimal growth conditions in contained industrial environments provides the ideal opportunity for (bacterio)phages to proliferate (2). Phages represent one of the most significant challenges to dairy fermentations as they cause production disruptions and may persist in the processing environment for extended time periods (3). Dairy streptococcal phages are classified into one of five genetically distinct groups: the *Moineauvirus* (formerly termed the *cos* group), *Brussowvirus* (formerly termed the *pac* group), *Vansinderenvirus* (formerly termed the 5093 group), 987 and P738 phages (4). In response to the persistent threat of phages, *S. thermophilus* strains have developed a collection of protective antiphage systems. It is widely understood that the defence systems of dairy streptococci predominantly include clustered regularly interspaced short palindromic repeats (CRISPR) and CRISPR-associated (Cas) genes as well as restriction and modification (R/M) systems (5,6). Furthermore, it has been shown that these systems work compatibly to increase the overall phage-resistance level of the host strain (7).

Beyond CRISPR-Cas and R/M systems, there are limited reports of other phage-resistance systems being active in *S. thermophilus*. These include the prophage-encoded lipoprotein Ltp system that was first described in *S. thermophilus* (pro)phage TPJ-34 (8). This system interferes with the process of phage DNA injection and was shown to provide resistance against both dairy streptococcal and lactococcal phages. Homologues of *ltp* have subsequently been identified in other *S. thermophilus* prophages and while the incidence of prophage carriage among strains of this species is reportedly low, it highlights the potential benefits conferred by integrated temperate phages (5,9). The phenomenon of phage-resistance systems providing protection in both streptococci and lactococci is not limited to this system. For example, the lactococcal abortive infection (Abi) system AbiA provides resistance against virulent phage infection in *S. thermophilus* strains at 30°C. Interestingly, this protective effect was abolished at temperatures at and above 37°C (10). Although the latter corresponds to the optimal growth temperatures for *S. thermophilus* in food fermentations, strains of this species will grow at 30°C and when applied as an adjunct in mesophilic fermentations, the activity of such cross-species functionality may present a considerable benefit to the robustness of the process.

In recent years, a wide range of novel phage-resistance systems have been identified in both Gram-positive and Gram-negative bacteria (11–15). The identification of so-called ‘Defence Islands’ on bacterial chromosomes has highlighted the presence of genomic regions dedicated to providing protection against phages (11,16,17). In *E. coli* many such defence hotspots were observed to be associated with mobile genetic elements and prophages (18). In parallel, the availability of bioinformatic tools to detect the presence of phage-resistance systems in bacterial genomes has significantly improved, in particular through tools such as PADLOC, MacSyFinder and Defensefinder (19–23). While *S. thermophilus* is a relatively young species (estimated 3000–30 000 years), its adaptation to growth in milk has resulted in extensive genome decay and horizontal gene transfer events has facilitated its diversification (24). Given the significant pressure imposed on strains of this species by phages, it is very likely that the consistent presence in fermentation facilities has selected for isolates that acquired genomic regions that encode phage-resistance systems. In the present study, the complete genomes of 27 *S. thermophilus* strains were sequenced using a Pacific Biosciences (PacBio) platform to evaluate the resistome landscape including functional analysis of a selection of predicted phage resistance systems and their functionality in both *Lactococcus cremoris* and *S. thermophilus*.

## Materials and methods

### Biological resources and culturing conditions

All *S. thermophilus* strains whose genomes were sequenced as part of this study are listed in Table 1. The strains that were selected for genome sequencing in this study are all dairy isolates and have been applied and/or characterized in previous studies (25–28). Furthermore, in a recent survey of the genetic diversity of the gene cluster associated with rhamnose-glucose polysaccharide (RGP) biosynthesis in *S. thermophilus*, the selected strains were shown to represent diverse genotypic groups (25). *S. thermophilus* Moz109 was furthermore used as a host for the evaluation of phage-resistance activity. *S. thermophilus* strains were grown in M17 broth (Oxoid Ltd, UK) supplemented with 0.5% lactose and incubated without agitation at 42°C for 16–18 h. *Lactococcus cremoris* NZ9000 was grown in M17 broth supplemented with 0.5% glucose and incubated at 30°C for 16–18 h without agitation.

**Table 1.** *S. thermophilus* strains applied in this study and their associated genome features

*S. thermophilus* strain	Genome size (Mbps)	GC % content	#CDS	Phage-resistance regions of genome [kb (%)]	Genbank accession no.
4021	1.861	39.07	1937	30.599 (1.6)	CP065384
4052	1.782	39.06	1883	23.994 (1.4)	CP065493
4067	1.821	39.01	1920	17.792 (1.0)	CP065476
4078	1.837	39.08	1944	28.536 (1.6)	CP065496
4134	1.855	39.08	1933	44.783 (2.4)	CP065477
4145	1.838	39.05	1931	35.111 (1.9)	CP065492
4147	1.797	39.08	1891	27.035 (1.5)	CP065504
90728	1.872	39.07	1959	41.833 (2.2)	CP065479
90729	1.877	39.14	1953	34.582 (1.8)	CP065480
90730	1.796	39.05	1883	25.360 (1.4)	CP065481
AVA1121	1.829	39.07	2003	29.506 (1.6)	CP065488
AVA116	1.737	39.21	1821	29.506 (1.7)	CP065498
CNRZ1151	1.794	39.05	1890	26.654 (1.5)	CP065483
CNRZ1202	1.79	39.09	1880	17.044 (1.0)	CP065506
CNRZ1575	1.823	39.21	1943	17.044 (0.9)	CP065490
CNRZ302	1.859	39.09	1975	33.663 (1.8)	CP065489
CNRZ385	1.897	39.07	1995	33.647 (1.8)	CP065495
CNRZ760	1.871	39.06	1959	31.448 (1.7)	CP065482
CNRZ887	1.78	39.13	1854	23.694 (1.3)	CP065491
MM1	1.843	39.1	1927	24.641 (1.4)	CP065484
MM20	1.942	38.81	2023	41.921 (2.2)	CP065485
ST128	1.854	39.12	1929	38.379 (2.0)	CP065500
ST19	1.863	39.01	1929	40.001 (2.2)	CP065487
ST1A	1.869	39.06	1947	29.721 (1.6)	CP065384
ST55	1.865	38.95	1964	36.626 (2.0)	CP065502
UCCSt95	1.794	39.19	1868	37.323 (2.1)	CP101646
R1	1.859	39.02	1951	26.701 (1.4)	CP065486

### Isolation of genomic DNA, sequencing, assembly and annotation

Genomic DNA was isolated from 27 *S. thermophilus* strains using the Macherey-Nagel NucleoBond system, utilizing Buffer Set III and AXG or AX 100 columns (Macherey-Nagel, Germany). This was performed according to the manufacturer's instructions with the following modifications: at the cell lysis step, the volume of lysozyme (100 mg/ml) added was increased to 40 μl, and 50 μl mutanolysin (5000 units/ml; Sigma-Aldrich, Germany) was also added prior to incubation, which proceeded for an increased period of 1 h. In addition, where necessary, insoluble components were removed from the supernatant by centrifugation up to 10 000 × *g* for 10 min prior to loading on the column. DNA quantity was measured by Qubit 2.0 (Life Technologies, USA) and quality assessed by visual inspection using agarose gel electrophoresis.

Genomic DNA was sheared to an average size of ∼10 kb using the G-tube protocol (Covaris, MA). DNA libraries were prepared using a SMRTbell express template prep kit 2.0 (100–938–900, Pacific Bioscience, CA, USA) and ligated with hairpin barcoded lbc adapters. Incompletely formed SMRTbell templates were removed by digestion with a combination of exonuclease III and exonuclease VII (NEB). The qualification and quantification of the SMRTbell libraries were made on a Qubit fluorimeter (Invitrogen, USA) and a 2100 Bioanalyzer (Agilent Technologies, USA). SMRT sequencing was performed using an SQ1 (Pacific Biosciences) instrument based on the multiplex protocol for 10 kb SMRTbell library inserts or Pacific Biosciences SMRT RSII technology. Raw sequencing reads were assembled using the hierarchical genome assembly process (HGAP) protocol RS_Assembly.2 in the SMRT analysis software v2.3 using the default settings for RSII sequence data. Sequencing reads emanating from the SQ1 sequencing instrument were *de novo* assembled using the Microbial Assembly version 10.1.0.1119588 (29) program with default quality and read length parameters. In addition to genome assembly, the SMRT Analysis pipeline from Pacific Biosciences (http://www.pacbiodevnet.com/SMRT-Analysis/Software/SMRT-Pipe) enables the determination of the epigenetic status of sequenced DNA by identifying m6A and m4C modified motifs (30–32).

Genome annotation was performed by NCBI using the PGAP annotation pipeline (33). Artemis (34) (v18) genome browser and annotation tool was used to inspect and, where necessary, to manually curate predicted ORFs. ORF annotations were refined where necessary using alternative databases including Interpro (35), HHpred (36) and Uniprot/EMBL (37). All sequence comparisons at a protein level were performed via all-against-all, bi-directional BLAST alignments. An alignment cut-off threshold of *E*-value <0.0001, with >30% amino acid identity across 80% of the sequence length was applied.

### Genome scan for potential phage-resistance systems and prophage regions

Primary scanning of the assembled genome for putative methyltransferase (MTase) genes was performed using the Seqware program (38). Additional searches for MTase genes were performed using HMMer (HMMER 3.3.2; http://hmmer.org/) to annotate each predicted protein coding sequence (CDS) feature in the genomes. Genes matching MTase sequence profiles were further examined by structure prediction using the ColabFold implementation of AlphaFold2 (39,40), followed by structure similarity search using predicted MTase models as a query inputs to DALI (41).

CRISPR-Cas encoding regions were identified using CRISPRCasFinder (https://crisprcas.i2bc.paris-saclay.fr/CrisprCasFinder/Index) and selecting the evidence level 2–4 outputs only (42). A general search for phage-resistance systems (including and beyond R/M and CRISPR-Cas systems) in the genomes of the 27 strains sequenced as part of this study was performed using PADLOC (https://padloc.otago.ac.nz/padloc/) using default settings (19). Where more than one strain was identified to encode similarly predicted phage-resistance systems (e.g. AbiD, AbiEi/AbiEii and Sirtuin-dependent system), the sequences of the similarly annotated systems were compared to each other using BLASTp analysis. Where a single example of a predicted phage-resistance system was identified (e.g. Hachiman, Gabija and Kiwa), a BLASTn search was performed against the NCBI database (https://blast.ncbi.nlm.nih.gov/Blast.cgi). To establish the distribution of phage-resistance systems in *S. thermophilus* strains’ genomes that are available in the RefSeq database, DefenseFinder was used with a search term of ‘Streptococcus thermophilus’ and visualizing the heat map under the species level of taxonomic rank (22). The distribution of systems was reported as the number of systems identified in the RefSeq database (from a pool of 462 RefSeq entries) while the heatmap reported the proportion of genomes presenting with a given system per species taxonomic rank.

Prophage regions were predicted using PHASTEST using default settings (43). Prophage regions are predicted by PHASTEST as intact, incomplete or questionable prophages. The identified regions were manually inspected to establish the gene content of the predicted prophages.

CR-Defence Islands were defined using the following criteria: (1) the CRISPR loci were used as the ‘anchor’ of possible defence islands and (2) the surrounding gene content was considered part of the defence island until conserved genes/gene clusters were reached and which were considered the outer boundaries of the proposed defence island. In this manner, three defence island regions were identified, CR1-Defence Island, CR2/4 Defence island and CR3-Defence Island.

### Construction of an R/M negative derivative of *L. cremoris* NZ9000

An *L. cremoris* NZ9000 mutant carrying deletion of a 5945 bp DNA fragment encoding a complete Type I R/M system (comprising *llnz_03 405*, *llnz_03 410* and *llnz_03 415* which encode HsdR, M and S, respectively) was constructed by double-crossover (DCO) recombination, using the plasmid pGhost9-ΔhsdRMS containing the *hsdRMS* flanking DNA fragments. To construct this plasmid, two PCR products of approximately 700 bp of the flanking regions of *hsdRMS* were amplified from NZ9000 using two pairs of primers (034RM1-034RM2 and 034RM3-034RM4, described in Supplementary Table S1) and Phusion high fidelity DNA polymerase (New England Biolabs). After purification, the PCR products were fused to thermosensitive plasmid pGhost9, digested with SmaI (New England Biolabs), by the strand overlap extension method, using the Gibson assembly cloning kit (New England Biolabs). The reaction mixture was introduced into *E. coli* JIM4646 with appropriate selection (Ery). For one such clone (which we named VES7817), the DNA sequence of the cloned fragment of pGhost9-ΔhsdRMS was verified by PCR and sequencing, using primers pGH9L and pGH9R (Supplementary Table S1). pGhost9-ΔhsdRMS was introduced to NZ9000 and presumptive transformant colonies were selected at the permissive temperature (30°C) on GM17 plates containing erythromycin (3 μg/ml; GM17 + Ery3). To select single cross-over events, a VES7817 overnight liquid culture (first grown in GM17 + Ery3 at 30°C) was plated on GM17 + Ery3 and incubated at the non-permissive temperature of 42°C. The second recombination event leading to plasmid excision was selected by inoculating resultant colonies in liquid GM17 medium without antibiotic at 42°C, and shifting exponentially growing culture to 30°C for 2 h, followed by overnight growth at 42°C. The culture was maintained on GM17 agar plates without antibiotic selection at 30°C. A strain harbouring a *hsdRMS* deletion (termed VES7862) was selected as an erythromycin-sensitive clone and the presence of the deletion in its chromosome was confirmed by PCR and sequencing using primers 034RmdelL and 034RmdelR (Supplementary Table S1).

### Cloning and confirmation of R/M activity

Cloning of selected R/M systems into the inducible vector pPTPi (44) was performed using conventional recombinant DNA techniques (oligonucleotide primers are listed in Supplementary Table S1). Induction of this promoter was achieved by the addition of nisin (5 ng/ml) in the growth medium. Obtained pPTPi constructs were subsequently transformed into the previously described R/M-free derivative *L. cremoris* VES7862. Confirmation of R/M activity of these pPTPi constructs was conducted by inducing each construct, and the empty vector control, with 5 ng/ml nisin and testing each strain for phage-resistance activity against the lactococcal *Skunavirus* sk1 and *Ceduovirus* c2 using an established double agar plaque assay method (45).

### Evaluation of phage-resistance activity of non-R/M, non-CRISPR systems

Representative genes that encode homologues of AbiD (*St55_0646, St4078_0760, St4021_1632*), AbiEi/AbiEii (*St90730_1386, St90730_1387*), Hachiman (*St19_0447, St19_0448*), Kiwa (*St90730_688, St90730_689*), Sirtuin-dependent system (*St19_0691*, *St19_0692*) and Gabija (*St4145_0685, St4145_0686*) were cloned into the high copy number plasmid pNZ44 under the control of the constitutive lactococcal P44 promoter (46). For systems incorporating predicted gene pairs, both genes were cloned in tandem. The primer pairs used for the amplification of the target genes are presented in Supplementary Table S1. The sequence of the generated constructs was verified by Sanger sequencing (Genewiz, Leipzig, Germany). The constructs were introduced into *L. cremoris* NZ9000, *S. thermophilus* 4078 and *S. thermophilus* Moz109 by electroporation. The empty vector was also introduced into the three strains as a control for phage assays. Phage-resistance activity elicited by the predicted phage-resistance systems was evaluated using plaque assays (45) and the efficiency of plaquing (E.O.P.) of phages was calculated by dividing the phage titre on the test strain (i.e. strains carrying the predicted phage-resistance system constructs) by the phage titre of the strain harbouring the empty vector. The *Skunavirus* sk1 and *Ceduovirus* c2 were used in plaque assays with the *L. cremoris* host strain NZ9000. The streptococcal *Brussowvirus* SW13 was used in plaque assays with *S. thermophilus* 4078 and the *Moineauvirus* STP1 was used in plaque assays with *S. thermophilus* Moz109. All assays were performed in (at least) triplicate and the presented results are the average of these data.

### Alphafold analysis of phage-resistance systems

Structure predictions of the identified putative phage-resistance protein(s) were performed with a Colab notebook running AlphaFold v2.3.1 (https://colab.research.google.com/github/deepmind/alphafold/blob/main/notebooks/AlphaFold.ipynb) or HPC resources from GENCI-IDRIS running AlphaFold v2.3.1 (40). The pLDDT values and the predicted alignment errors (PAE) were obtained from the Colab Notebook or the IDRIS calculations output. The pLDDT values were also stored in the B-factor column of the PDB files. The pLDDT and PAE plots for each protein of complex are provided as Supplementary Figures S1 and S2. The final predicted protein or domain structures were submitted into the Dali server (41) to identify the closest structural homologs in the PDB. Dali provides a root mean square deviation value (r.m.s.d.) in Å, as well as an aggregated factor called *Z*-value. A *Z*-score above 20 means the two structures are definitely homologous, between 8 and 20 means the two are probably homologous, between 2 and 8 is a grey area, and a *Z*-score <2 is not significant. Visual representations of the structures were prepared with ChimeraX (47). The ‘rainbow’ color coding consist of applying rainbow colors to the protein ribbon representation from blue, at the N-terminus, to red at the C-terminus. Proteins superpositions were performed using Dali superpositions (41).

## Results and discussion

### *S. thermophilus* dedicates a significant portion of its genome to phage defence

The genomes of 27 *S. thermophilus* strains were sequenced to completion. The genomes were shown to possess a G + C content of approximately 39%, are between 1.78 and 1.94 Mb in length and are predicted to harbour 1821 to 2023 coding sequences (Table 1). These *S. thermophilus* genomes were analysed for the presence of phage-resistance systems using PADLOC. The combined length of the genetic regions that are dedicated to the predicted phage-resistance systems (excluding phage defence candidate systems) was calculated and ranged between 17 and almost 45 kb representing 0.9–2.4% of total bacterial genetic content. These genomic regions primarily comprise of genes encoding CRISPR-Cas (0.2–1.3% of total genome) and R/M (0.1–0.7% of total genome) systems; however, homologues of lactococcal abortive infection (Abi) and more recently identified phage defence systems from other bacteria were also identified in the analysed genomes with further details presented below. Based on the current analysis, strains of *S. thermophilus* are predicted to harbour an approximate average of seven phage-resistance systems per genome with CRISPR-Cas and R/M systems representing their core phage resistome and incorporating additional and, in many cases, strain-specific repertoires of phage-resistance systems.

In addition to the chromosomally-located phage defence systems, we evaluated the contribution of mobile genetic elements to the anti-phage repertoire in this species using PADLOC. *S. thermophilus* strains do not typically harbour many, if any, plasmids, likely owing to the presence of CRISPR-Cas systems in strains of this species. Among the strains analysed in this study, plasmids were identified associated with strains ST1A, 4021, 4067, 4147, CNRZ1202 (2 plasmids), AVA116, ST55 and ST128 (Supplementary Table S2). These plasmids range in size from ∼3.4 to 8.2 kb and are predicted to harbour between two and seven ORFs (Supplementary Table S2). Of the nine identified plasmids, only one possessed an identifiable (complete) anti-phage system, i.e. a Type II R/M system on pCNRZ1202A. Additionally, the genomes were analysed for the presence of prophage-associated regions and their possible contribution to the phage-resistance landscape. Using the outputs of Phastest, 12 of the 27 assessed *S. thermophilus* genomes were predicted to have at least one prophage region, with one genome (i.e. that of strain CNRZ385) possessing two predicted ‘questionable’ prophage regions (Table 2). Among the identified prophage regions, only one possesses an identifiable superinfection exclusion system, which may contribute to phage-resistance in the host strain (STR1). Nonetheless, all identified prophage regions harbour several transposase-encoding genes, highlighting the importance of these regions as possible recombination sites and repository for novel genetic acquisitions. Therefore, it appears that mobile genetic elements including prophages and plasmids are not a major source of anti-phage systems while chromosomal variations and acquisitions are dominantly associated with the defences of this species.

**Table 2.** CRISPR-Cas profiles* and prophage characteristics of *S. thermophilus* strains with number of spacers/prophages indicated. Grey shaded boxes indicate absence of features

*Sth* strain	CR1	CR2	CR3	CR4	# Prophage regions predicted	Size (kb); location
4021	14		16		1 questionable	40.7; 768552–809322
4052			24			
4067			7		1 incomplete	32.6; 1649507–1682124
4078	20	1	36		1 intact	16.2; 960763–977025
4134	12	3	32	12	1 questionable	37.7; 761384–799143
4145	19	1	30		1 questionable	37.7; 752479–790239
4147		1	14			
90728	16	4	10	18		
90729		3	11			
90730		1	29			
AVA1121	16		9			
AVA116		7	18			
CNRZ1151		1	29			
CNRZ1202			23			
CNRZ1575	25		30			
CNRZ302	17	1	24			
CNRZ385	18	1	20		2 questionable	37.7; 766659–804403
						23.7; 830040–853786
CNRZ760	35	1	28			
CNRZ887		4	29			
MM1		1	24			
MM20		16	23	20		
STR1	23		17		1 intact	37.1; 731756–768888
ST128	46	1	22		1 questionable	59.4; 23992–83425
ST19	17	3	37		1 questionable	37.8; 754480–792319
ST1A	16	3	16		1 questionable	40.7; 764773–805543
ST55	23	3	17			
UCCSt95	28	1	27		1 questionable	37.6; 729101–766773

### The CRISPR-Cas landscape of *S. thermophilus*

**Figure 1.**
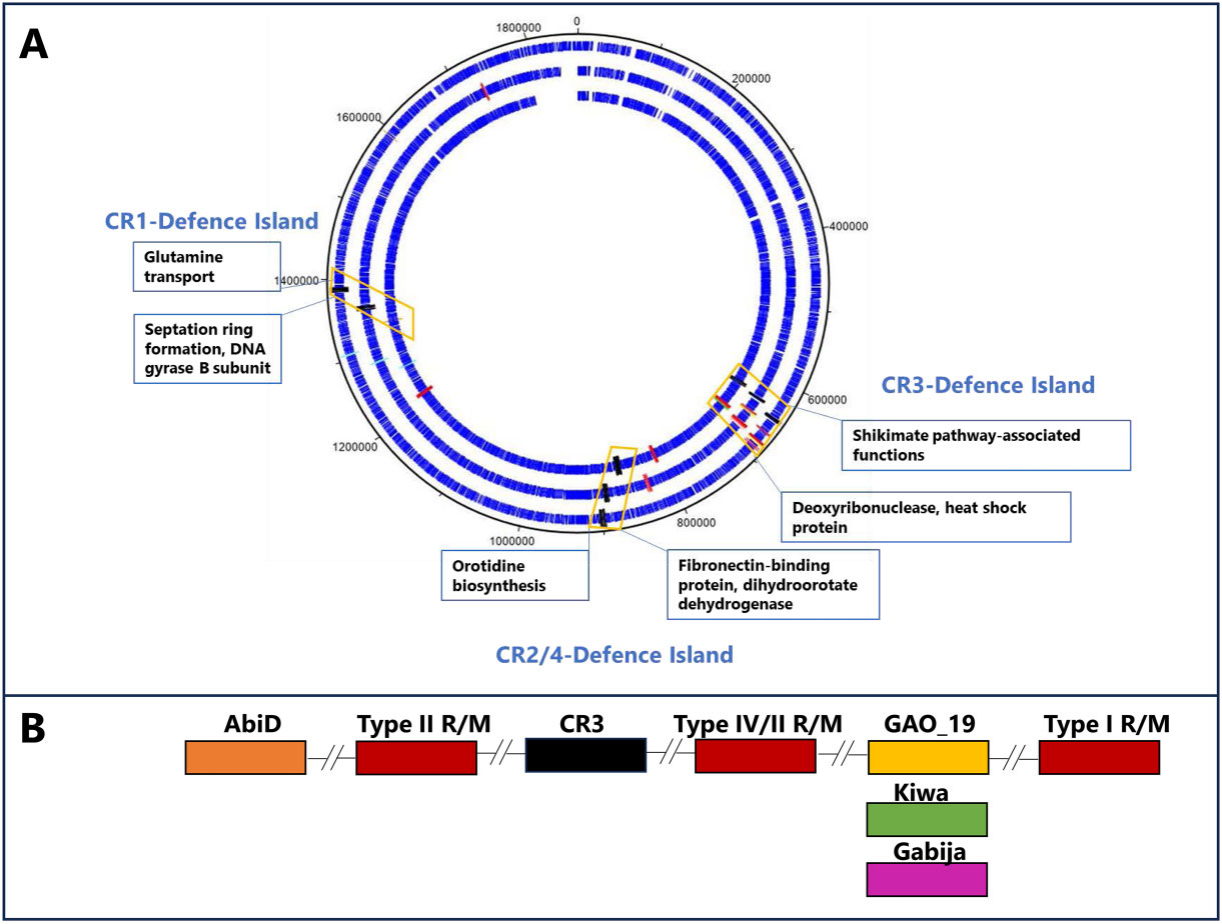
**(A)** DNAPlotter view of chromosomes of *S. thermophilus* 4021 (outside track), 4145 (middle track) and 90730 (inside track) with CRISPR-Cas regions indicated in black, R/M systems (red), Abi systems (D or E; orange), PD-T4-6 (light blue, all), GAO_19 (yellow, 4021 and 4145), Kiwa (green, 90730) and Gabija (pink, 4145) highlighted in the relevant genomes where they occur. The CR-associated Defence Islands are highlighted in orange boxes with the annotated functions of flanking regions presented in blue boxes. (**B**) CR3-Defence Island harbours CR3, R/M system(s) and Gabija, Kiwa, GAO_19 and certain AbiD-encoding genes, where they occur.

Strains of *S. thermophilus* are reported to harbour up to four CRISPR-Cas systems, referred to herein as CR1 through to CR4. CR1 and CR3 are classified as Type II-A and Type II-C systems, respectively, and are the most widely reported functional systems in this species (48). CR2 and CR4 are classified as Type III-A and Type I-E systems, respectively (1). The genomes of the 27 strains used in this study were scrutinized for the presence of CRISPR-Cas systems using CRISPRCasFinder, confirming the ubiquity of CR3 systems among the 27 analysed strains (Table 2). CR1 and CR2 were identified in 16 and 20 of the 27 genomes, respectively, while CR4 was present in just three genomes. Typically, CR2 systems are observed to incorporate/retain the lowest number of spacers, while CR3 and CR1 incorporate or retain considerably higher numbers of spacers perhaps reflecting the activity level of these systems. CR4 systems present in the genomes of strains 4134, 90728 and MM20 were observed to harbour 12, 18 and 20 spacers, respectively, which also suggests that these systems are functional and active (Table 2). The genetic regions specifying CR1, CR2 (where present) and CR3 CRISPR-Cas regions are located in conserved genomic positions in all analysed strains with the CR1 locus positioned between genes associated with orotidine biosynthesis, the CR2 locus (when present) located downstream of genes associated with septation ring formation and the CR3 locus located downstream of shikimate pathway-associated functions (Figure 1A).

### The *S. thermophilus* methylome is dominated by type I systems

**Figure 2.**
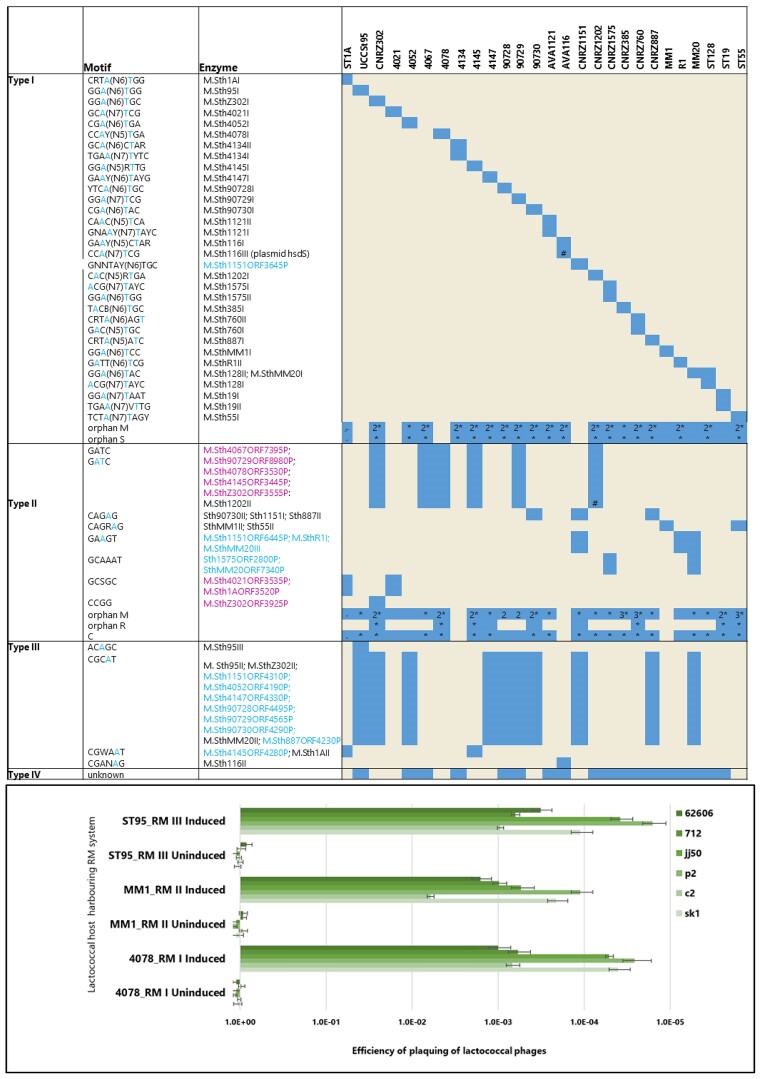
Upper panel: Identified R/M systems (both active systems and inactive systems/individual genes). Methylated bases within the detected motifs based on SMRT sequencing are indicated in blue text in the ‘motif’ column. Predicted m5C (pink text) and m6A (blue text) associated enzymes which were predicted but where methylation activity was not detected in this study. Where more than one gene was identified, the number of such genes is indicated by a number in the relevant box. In the ‘Enzyme’ column, black text indicates enzymes for which associated methylated motifs were identified, blue and pink text indicates enzymes for which m6A and m5C recognition sequences, respectively, were predicted but methylated motifs were not detected. Lower panel: Efficiency of plaquing (EOP) of lactococcal phages in the presence (induced) or absence (uninduced) of Type I, Type II or Type III R/M systems. In the uninduced state, there is no observable phage-resistance while in the induced state, all three evaluated R/M systems were observed to elicit substantial resistance against the evaluated phages 62606, 712, jj50, p2, c2 and sk1.

The products of all identified ORFs of the genomes were compared by BLASTp alignments against the REBASE database (http://rebase.neb.com/rebase/rebase.html) to identify potential R/M system genes. Using this approach 78, 60, 14 and 18 predicted Type I, II, III and IV R/M systems were identified (albeit with apparent frameshifts in some) (Figure 2; Supplementary Tables S3-S11). PacBio-derived genome sequence data sets were analysed to identify DNA methylation motifs, and the genes for each methylase were matched with these motifs wherever possible. In many cases, a predicted restriction enzyme-encoding gene was located next to or close to an active methylase, indicating that a complete and active R/M system is present. Among the 27 analysed genomes, 26 (all except strain 4067) were shown to possess methylated motifs, which were solely represented by m6A base modifications. In this manner, a total of 49 distinct methylated motifs were identified, among which 32 are attributable to Type I, 11 to Type II and 6 to Type III R/M systems (Figure 2; Supplementary Tables S3-S5).

In addition to the 49 methylated motifs, each of which could be assigned to a specific R/M system as based on alignments against the REBASE database, 29 Type I R/M system genes were identified that do not have a corresponding methylated motif, often due to frameshift mutations that rendered the system (perhaps temporarily) inactive (Supplementary Figures S6). Furthermore, five strains harbour an m5C Type II R/M system associated with a GATC recognition sequence, two associated with a GCSGC recognition sequence and one with a CCGG recognition sequence (Figure 2; Supplementary Tables S7 and S8). Methylation was not detected for these systems in the present study, most likely owing to the technological approach used (which does not accurately detect cytosine-specific methylations); however, it is likely that these systems are functional. This highlights the presence of possible cytosine-specific methyltransferases in this species in addition to the dominant adenine methylase activity (Figure 2). All known Type III R/M systems to date are described to (hemi)methylate an adenine (m6A or m4C) (49). While the Type III R/M system associated with a CGCAT motif was identified in the genome of ten strains, methylation of this motif was only observed in the genomes of three strains i.e. CNRZ302, MM20 and UCCSt95 (Figure 2). The apparent non-functionality of this (and other) R/M systems appears to be due to observed frameshifts in the associated gene(s) (Supplementary Table S8). Type IV systems do not encode a methylase and typically cleave methylated or unusually modified (hydroxymethylated or glucosyl-hydroxymethylated) bases (50) and may not be detected using the approach applied in this study (Figure 2 and Supplementary Table S10).

### Restriction & modification systems are encoded by genes that occur in proximity to CRISPR loci

Overall, the 27 assessed *S. thermophilus* strains are predicted to encode between one and five active R/M systems with an average of 3.4 R/M systems per strain. Type I R/M systems were shown to be encoded by genes that occur at several distinct genomic locations. Among the genetic determinants of the Type I systems analysed, the majority (24 of 33) are located in a conserved location downstream of CR3 and to a lesser extent, in proximity to CR2. The genes encoding identified Type II and Type III R/M systems occur in conserved locations upstream or downstream of CR3. The Type IV systems are encoded by genes that typically occur downstream of CR3 systems (in 17 out of 18 cases). In the case of strain MM20, genomic rearrangements are evident although the genomic context of the predicted Type IV remains the same as for the other strains while in contrast, the Type IV system of strain CNRZ385 is present in a region rich in transposase elements, which may have facilitated genomic inversions and which likely accounts for its unusual genomic location relative to the other strains. While the vast majority of R/M systems identified in this study are chromosomally-located, the enzymes responsible for two methylated DNA motifs appear to be plasmid-encoded, i.e. *S. thermophilus* AVA116 (motif CCA(N7)TCG) and *S. thermophilus* CNRZ1202 (motif GATC) (Figure 2). In AVA116, a solitary *hsdS* specificity subunit is present in the plasmid pAVA116A that is expected to interact with the chromosomal Type I methylase subunit to methylate its recognition sequence. Interestingly, PADLOC analysis did not identify this hsdS subunit, which may highlight the importance of methylation analysis and tailored searches for R/M system components to establish the complete methylome. The plasmid-associated Type II system of *S. thermophilus* CNRZ1202 is a complete system encoded by genes on the plasmid pCNRZ1202A (Supplementary Table S2).

While the methylase functionality of the m6A systems was established through methylation profiling based on PacBio SMRT sequence data, it was decided to establish the functionality of representative cognate restriction enzymes in terms of providing phage resistance. This was achieved by cloning genes encoding a representative Type I (from *S. thermophilus* 4078; motif CCAYN_5_TGA), II (from *S. thermophilus* MM1; motif CAGRAG) or III (from *S. thermophilus* St95; motif ACAGC) R/M system in the low copy vector pPTPi, after which the resulting constructs were introduced into the R/M-free lactococcal strain *L. cremoris* VES7862 by electroporation. Molecular tools to modify the genomes of *S. thermophilus* are not as well developed as for some bacterial species (such as *L. cremoris*), while the genetic amenability of individual *S. thermophilus* strains is also unpredictable and often poor. Therefore, functional validation of the selected R/M systems was performed in *Lactococcus cremoris* due to the availability of genetic tools to generate a R/M-free derivative of the test strain and the similar GC % content of *Lactococcus* and *S. thermophilus*. The R/M system-carrying strains were evaluated for their ability to reduce the efficiency of plaquing of the lactococcal skunaviruses sk1, p2, jj50 and 62606 and the *Ceduovirus* c2. All three heterologously expressed R/M systems elicited a clear phage-resistance phenotype (three to five log reduction in E.O.P.) against all tested phages confirming the functionality of the selected R/M systems’ restriction enzymes in addition to the defined methylase function (Figure 2; Supplementary Table S12). Cross-species functionality of R/M (and other phage-resistance) systems between mesophilic lactococci and thermophilic dairy streptococci has previously been reported. Specifically, the Type II R/M system LLaII (which recognizes a GATC target sequence) identified on the lactococcal plasmid pSRQ700 was found to be functional at 42°C in *S. thermophilus* when provided *in trans* on a suitable replicating vector (51).

### *S. thermophilus* defence islands

Several studies have reported the co-location of phage-resistance systems in so-called ‘Defence Islands’ in both Gram-negative and Gram-positive bacteria (11,15,17). Furthermore, analysis of the CRISPR-Cas and R/M systems in this study, identified that these are most often located in conserved locations in dairy streptococcal genomes and in relative proximity between certain CRISPR-Cas and R/M systems. Therefore, it was considered that these regions constitute Defence Islands in *S. thermophilus* and represent locations to identify additional phage-resistance systems. Since CRISPR-Cas systems are always present in *S. thermophilus* genomes, we chose to use the CR1, CR2 and CR3 regions as genomic beacons for these Defence Islands. Herein, the Defence Islands are named in accordance with the CR region with which they coincide and will be discussed in order of appearance in the genomes of the analysed strains, i.e. CR3-Defence Island, CR2/4-Defence Island and CR1-Defence Island (Figure 1A). CR4 regions were identified in the genomes of just three strains and these are located in relative proximity to CR2 systems and would thus be considered part of the CR2/4-Defence Island. Using the outputs of the PADLOC analysis mentioned above, the location of the identified non-CRISPR and non-R/M phage-resistance systems was evaluated in the context of these three proposed Defence Islands. Additionally, the prophage regions identified in the analysed genomes were evaluated for their proximity to CR-Defence Islands. Among the identified prophage regions, eight are in a similar relative position on the genome between the CR3- and CR2/4-Defence Islands. These prophage regions are ‘questionable’ or ‘incomplete’ prophage regions and primarily incorporate transposases, an integrase-encoding gene, a structural protein-encoding gene and genes predicted to encode nucleoside phosphorylases across regions spanning ∼37–41 kb. Also included in this cohort is one predicted intact prophage (in strain R1’s genome) although manual inspection did not identify an obvious morphogenesis module rendering it unlikely to form infective phage particles. The location of these prophage regions (although seemingly cryptic prophage regions) between the CRISPR loci combined with the density of transposase-encoding genes in this region highlights the likely genomic plasticity of this region among *S. thermophilus* strains. The remaining four prophage regions are in a variety of genomic positions across their hosts’ genomes without identifiable phage-resistance systems identified within them.

### CR3-defence Island- a hotspot of abortive infection anti-phage systems

**Figure 3.**
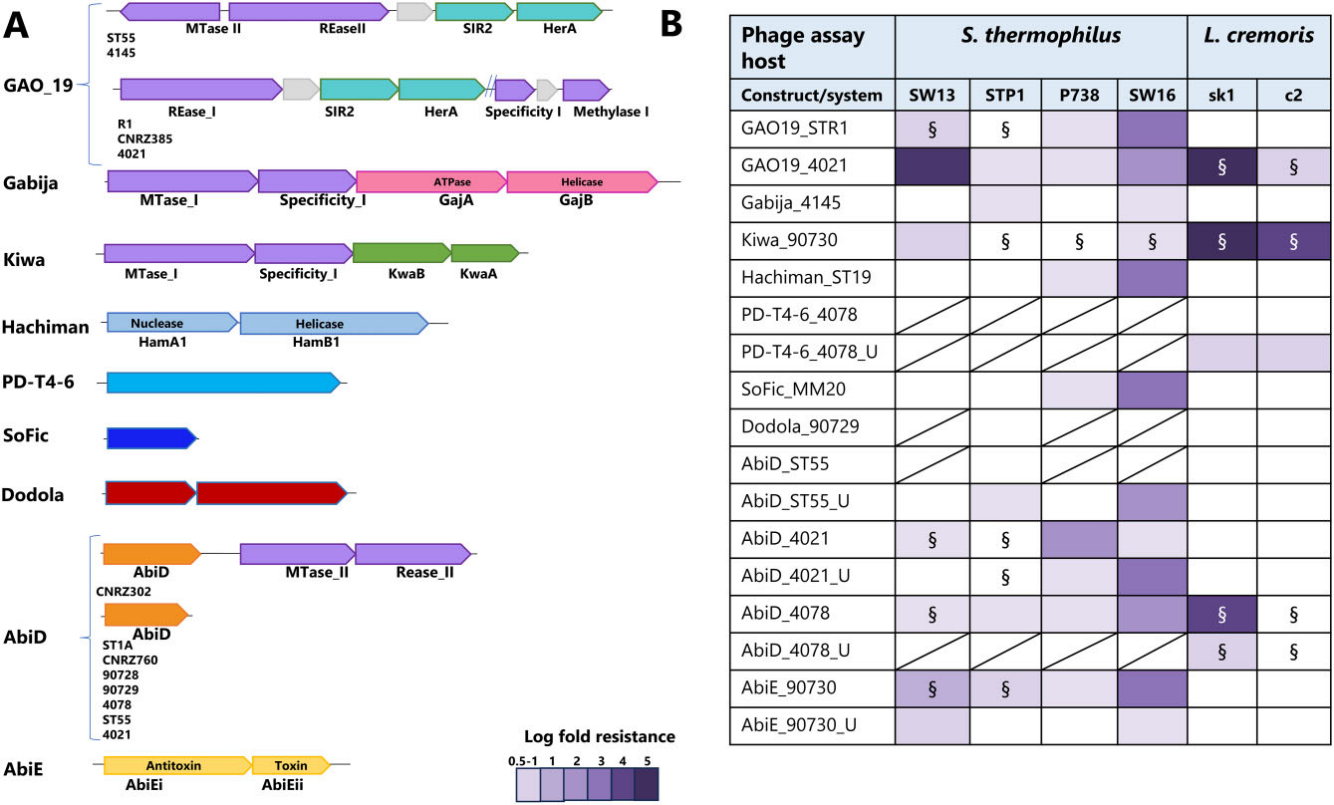
(**A**) Schematic depiction of the gene(s) associated with the identified anti-phage systems and their co-location with R-M systems, where relevant. (**B**) Phage resistance level provided by the evaluated anti-phage systems tested against phages that infect *S. thermophilus* (*Brussowvirus* SW13; *Moineauvirus* STP1; P738 namesake and; 987 group phage SW16) and *L. cremoris* (*Skunavirus* sk1 and *Ceduovirus* c2). The log fold resistance is colour-coded according to the colour scale bar at the bottom left of the table. § indicates where the average plaque size reduction was observed in the presence of the anti-phage system relative to the wild type strain.

CR3-Defence Island is the largest of the three defence islands with an average size of 74.5 kb (ranging from 51.0–128.5 kb) incorporating on average 77 predicted genes (ranging from 62–131 genes). This island is flanked by a conserved gene cluster associated with the shikimate pathway at the 5′ end and a conserved heat shock protein-encoding gene at the 3′ end. Between the genes encoding these functions are a plethora of (putative) anti-phage systems including CR3 (all strains) as well as Type I, Type II and/or Type IV R/M systems in conserved locations relative to CR3 (Figure 1B). Within this region, there are several highly conserved genes (>90% sequence similarity) with predicted functions including manganese transport, proteases, biotin synthesis and histidine protein kinase, among others. Approximately 25 genes of mostly unknown function represent the variable gene content in the overall CR3-Defence Island. Among these variable genes of the CR3-Defence Island, PADLOC analysis identified genes encoding predicted AbiD, GAO_19, Kiwa and Gabija in a small number of strains (Table 3; Figures 1B and 3A).

**Table 3.** Phage-resistance systems beyond CRISPR-Cas and R/M identified by PADLOC. Bold face text indicates the systems selected for functional evaluation

Strain	PADLOC identified phage-resistance systems	ORF number	Location on genome
ST1A	AbiD	1639	1601461–1602441c
	PD-T4-6	1341	1312938–1314809c
	PDC-S29	1815	1760204–1760938c
UCCSt95	PDC-S07	0654	642989–643378
	PD-T4-6	1285	1259864–1261735c
CNRZ302	AbiD	03920	760545–761435
	PD-T4-6	1370	1309158–1311029c
CNRZ385	GAO_19 (SIR2, HerA)	0755, 0756	691015–693872
	PD-T4-6	1417	1319589–1321460c
CNRZ760	AbiD	0656	641609–642619
	PD-T4-6	1366	1312221–1314092c
CNRZ887	AbiEi/Eii	1349, 1350	1311759–1312675
	PD-T4-6	1277	1239802–1241673c
CNRZ1151	AbiEi/Eii	1395, 1396	1334306–1335222
	PD-T4-6	1328	1271059–1272930c
90728	AbiD	0633	621588–622598
	PD-T4-6	1371	1323778–1325649c
90729	AbiD	0642	633131–634141
	PD-T4-6	1342	1313302–1315173c
	**Dodola (*dolA, dolB*)**	**1798, 1799**	1746878–1748836c
90730	**AbiEi/Eii**	**1386, 1387**	1336150–1337066
	**Kiwa (*kwaB, kwaA*)**	**0688, 0689**	673224–674776
	PD-T4-6	1310	1262508–1264379c
STR1	**GAO_19 (*SIR2*,*HerA*)**	**0691, 0692**	681184–684217
	PD-T4-6	1358	1311954–1313825c
ST19	**Hachiman type I**	**0447, 0448**	453847–457022
	PD-T4-6	1322	1295405–1297276c
ST55	**AbiD**	**0646**	624941–625951
	PDC-S58	0671	651314–652729
	GAO_19 (*SIR2, HerA*)	0721, 0722	698303–701093
	PDC-S06	0741	716435–718624
	PDC-S61	0744	720509–721261
	PD-T4-6	1380	1327282–1329153c
ST128	PD-T4-6	1338	1305964–1307835
4021	**GAO_19 (*SIR2, HerA*)**	**0687, 0688**	681458–684306
	**AbiD**	**1632**	1594135–1595115 c
	PD-T4-6	1335	1305732–1307603c
4052	PDC-S07	0664	643370–643759
	PD-T4-6	1298	1239930–1241801c
	AbiEi/Eii	1371, 1372	1309955–1310871
4078	**AbiD**	**0760**	741877–742767
	**PD-T4-6**	**1344**	1286907–1288778c
4134	PD-T4-6	1337	1300554–1302425c
4145	**Gabija (*gajA, gajB*)**	**0685, 0686**	679404–682765
	GAO_19 (*SIR2/HerA*)	0663, 0664	657103–659392
	PD-T4-6	1313	1283833–1285704c
4147	AbiEi/Eii	1393, 1394	1337577–1338493
	PD-T4-6	1317	1263929–1265800c
MM1	PD-T4-6	1333	1295884–1297755c
MM20	PD-T4-6	0620	604802–606673
	**Sofic**	**0248**	248143–248967
	Sofic	1073	1051268–1052092c
AVA116	PD-T4-6	1250	1210550–1212421c
AVA1121	PD-T4-6	1339	1276199–1278070c

Defence-associated sirtuins (DSRs) have been reported to deplete NAD^+^ through the NADase activity of SIR2 (called GAO_19 systems) and exhibit an Abi phenotype (52). Abi is a defense strategy where the infected cell commits suicide before the phage can complete its replication cycle, thereby protecting the remaining kin cell population and are particularly effective at low multiplicities of infection i.e. Abi phenotypes are typified by strain collapse in the presence of phages at high multiplicities of infection (MOI) but not where low MOIs are applied. For their activity, GAO_19 systems require a second protein, which differs depending on the host species, i.e. the DNA translocase HerA or pAgo. Bacterial DSRs of *Bacillus subtilis* have recently been demonstrated to recognize the phage tail tube leading to the activation of SIR2-mediated NAD^+^ hydrolysis (53). The chromosomes of *S. thermophilus* STR1, CNRZ385, ST55, 4145 and 4021 harbour homologues of the SIR2-dependent NAD^+^ depletion system encoded by *sir2/herA* (Table 3). The encoded SIR2/HerA system of CNRZ385 and 4021 are almost identical to each other (SIR2: 100% identity; HerA: 99% identity over 88% of the protein sequence) as are those of strains 4145 and ST55 (SIR2: 96.3% identity; HerA: 97.8% identity). The associated gene pair is in a similar position in the genome of each of the carrying strains (Figure 1A & B). Conversely, the SIR2/HerA system of STR1 displays markedly reduced sequence similarity to those of the other four strains. The GAO_19 SIR2/HerA systems are observed to be inserted between (R1, CNRZ385 and 4021) or downstream of (ST55 and 4145) R/M systems in CR3-Defence Island (Figures 1B and 3A). The GAO_19 *sir2/herA* gene pairs of R1 and 4021 were cloned in pNZ44 as representatives of the two genotypes of this system and transformed into lactococcal and streptococcal host strains to establish their ability to confer phage-resistance. GAO_19_4021_ provides resistance against sk1 and a moderate level of protection against SW13 and c2, while GAO_19_STR1_ provides up to three logs of protection against three of the tested streptococcal phages (Figure 3B).

**Figure 4.**
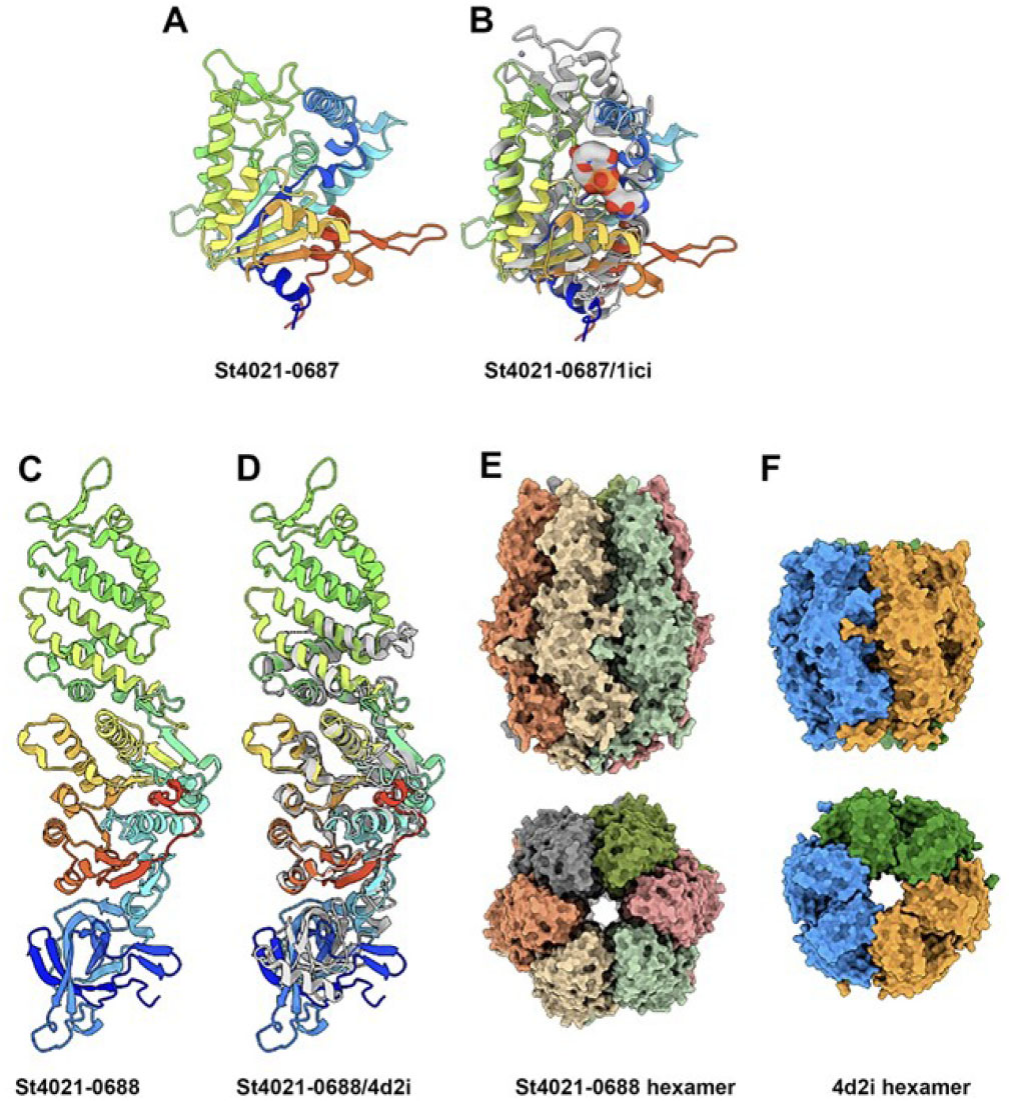
Structure prediction of the SIR2-HerA proteins of the GAO_19 system. (**A**) Ribbon structure of 4021–0687 (rainbow colored). (**B**) Superposition of 4021–0687, SIR2_4021_ (rainbow colored) with PDB 1ici (grey), a NAD-dependent protein deacetylases (*Z* = 14.0, rmsd = 3.3Å). with the NAD residue (in sphere mode) from 1ici in the catalytic crevice. (**C**) Ribbon structure of the three domains 4021–0688, HerA_4021_ (rainbow colored). (**D**) Superposition of 4021-0688 (rainbow colored) with PDB 4d2i (grey), a HerA ATPase from *Sulfolobus solfataricus* (*Z* = 29.4, rmsd = 4.2 Å). (**E**) molecular surface representations (side and top views) of 4021–0688 hexamer compared to (**F**) the molecular surface of the HerA ATPase from *Su. solfataricus*. A–F): the ‘rainbow’ color coding consists of applying rainbow colors to the protein ribbon representation from blue (N-terminus) to red (C-terminus).

SIR2 proteins are well characterized in eukaryotes and have been implicated in transcriptional silencing, cell cycle progression and genome stabilization functions (54). All SIR2 homologues studied to date possess NAD-dependent deacetylase activity highlighting the central role of this activity to the functionality of the system (55–57). The structures of the GAO_19 associated sirtuin-dependent systems identified in this study (ST4021-0687 (SIR2) and ST4021-0688 (HerA)) were predicted with AlphaFold2 (Figure 4A-D; Supplementary Figures S4E–G). SIR2_4021_ has a compact α/β-fold. Dali analysis of SIR2_4021_ identified hits with NAD-dependent deacylase (PDB 4twi-A) and a transcriptional regulatory protein of the SIR2 family (PDB 1ici). The predicted structure of SIR2_4021_ superimposes well on the SIR2 of PDB 1ici (Figure 4B). HerA_4021_ is a three-domain protein that exhibits structural similarity to and superimposes well on HerA (PDB 4d2i; Figure 4C, D) based on Dali analysis. Structure prediction of a hexamer of HerA_4021_ produces a model with a high confidence level similar to HerA (PDB 4d2i). In *Thermus thermophilus* this protein is defined as possessing ATPase activity whose structure comprises a conically-shaped, double hexamer ring with a central pore, consistent with our predictions (Figure 4E and F) (58).

Gabija was first identified in *Bacillus cereus* and was observed to be active against Myo-, Sipho- and Podophages and was found in 8.5% of microbial genomes tested (17). This is a two protein sensor-effector system incorporating GajA, an ATPase with DNA nicking activity, and GajB, a helicase, which together form a ∼500 kDa DNA-degrading complex (59). GajB senses DNA termini leading to nucleotide hydrolysis and the concomitant nucleotide depletion culminates in cellular abortion (14,60). In contrast to the broad spectrum of activity of previously described Gabija systems, no significant phage-resistance was elicited against the tested phages by Gabija_4145_ with the exception of a very modest antiphage activity (0.5–1 log reduction in EOP) against STP1 and SW16 (Figure 3B). The Gabija system identified in the genome of 4145 is encoded by *ST4145_0685* and *ST4145_0686*. AlphaFold prediction of the Gabija proteins 3D structure is consistent with previous functional analysis in which GajA yielded a three-domain protein that assembles as a dimer (Supplementary Figures S1A, B, S4H) and with Dali hits with an ATP-dependent endonuclease (PDB 6p74) (Supplementary Figures S1C). GajB_4145_ was predicted as a compact α/β-protein that yielded Dali hits against DNA helicases (PDB 4c2u; 4c30; 4c2t) (Supplementary Figures S1D, E, S4I). Gabija is a seemingly rare system among *S. thermophilus* strains as BLASTn analysis of *ST4145_0685* and *ST4145_0686* against the NCBI database identified just a single homologue of this gene pair in *S. thermophilus* ST64987 (*E*-value 0.00).

Kiwa incorporates *kwaA/kwaB* (Figure 3A) and is present in 1.8% of microbial genomes evaluated previously (17). The encoded system is described as a membrane-associated system that is triggered by the inhibition of host RNA polymerase and interferes with phage replication through a RecBCD-dependent pathway (61). While KwaB exerts an anti-phage effect independently of KwaA, it is believed that KwaA is required for its controlled expression (61). KwaB is predicted to be a two-domain protein with a α /β-N-terminal domain and an all α-helical C-terminal domain based on AlphaFold analysis (Supplementary Figures S1F, S4J). KwaB is predicted to dimerize primarily through its N-terminal domain (lower parts of structure in Supplementary Figures S1G, S4L). KwaA was predicted as an extended mainly α-helical protein (Supplementary Figures S1H, S4K) and produced hits against the *E. coli* stress protein YciF (PDB 2gs4; Supplementary Figures S1I) (62). Kiwa_90730_ exhibits a broad spectrum with strong anti-phage activity against lactococcal phages sk1 and c2 (5- and 4-log reduction of plaquing ability, respectively) and rather modest (1 log) resistance and plaque size reduction against tested streptococcal phages (Figure 3B and Supplementary Table S13).

AbiD-encoding homologues were identified in the chromosome of eight strains, i.e. *S. thermophilus* ST1A, CNRZ302, CNRZ760, 90728, 90729, ST55, 4021 and 4078 based on BLASTn searches in the NCBI database using default settings (Figure 3A). BLASTp comparisons of the eight AbiD proteins identified three distinct sets based on the level of sequence similarity, i.e. AbiD encoded by ST55, 90728, 90729, and CNRZ760; AbiD of CNRZ302 and 4078; and AbiD of 4021 and ST1A. Those identified in strains ST55, 90728, 90729 and CNRZ760 are all associated with a large genomic insertion flanked by several transposase-encoding genes that essentially doubles the size of this Defence Island in the genomes of these strains. The insertion is conserved across these four strains and includes genes with predicted products including bacteriocin biosynthesis, SOS response protein UmuC and heavy metal resistance functions. The AbiD proteins encoded by members of each of the three groups are identical within the groups while between groups, the sequence identity drops to ∼25% across approximately half of the compared amino acid sequence. Six of the eight AbiD-encoding homologues are located within CR3-Defence Island. In the genome of CNRZ302, the AbiD-encoding gene is directly upstream of a Type II R/M system (Figure 3A).

Alphafold2 analysis of representative AbiD proteins (those encoded by ST55, 4078 and 4021) established significant structural conservation of these proteins despite their low levels of sequence identity (Supplementary Figures S2A–C, S4A–C). Furthermore, superimposition of these three proteins with a lactococcal AbiD protein (Supplementary Figures S2D, S4D). highlights the maintenance of structural similarity of AbiD across these distinct species despite amino acid sequence similarities of approximately 5% (Supplementary Figures S2D-F). Furthermore AbiD-ST55, as an example, shares structural homology with the core of PDB ID 6iv8 (residues 163–330 and 491–576), a Cas13d ribonuclease (Supplementary Figure S2E). BLASTn analysis of AbiD-encoding genes revealed identical homologues in the genomes of nineteen strains of *S. thermophilus* in the NCBI database, highlighting the broad presence of this system among dairy streptococci. The evaluated AbiD systems were observed to provide low to moderate levels of protection against the streptococcal phages, while only AbiD_4078_ was observed to exert an effect against assessed lactococcal phages (Figure 3B).

### CR2/4 and CR1-Defence Islands rely on innate and adaptive immune systems

The CR2/4-Defence Island is demarcated by genes associated with orotidine biosynthesis flanking a variably present CR2 region and in two strains a CR4 region. This island spans approximately 17.5 kb (ranging from 15.9 to 18.7 kb) and typically contains ∼20 open reading frames among which an average of five predicted genes have no assigned functions. This region is quite conserved among *S. thermophilus* strains although strain-specific genes are present among the unassigned genes. While CR2 and CR4 are less prevalent when compared to CR3 and CR1, the CR2/4-Defence island remains an interesting region to analyse for the presence of novel anti-phage systems since Type III R/M systems are always (and Type I R/M systems occasionally) located upstream of this region. Furthermore, the region upstream of the CR2/4-Defence Island is rich in IS-elements and transposase-encoding genes and predicted prophage regions in eight of the analysed genomes (as described above) while the highly variable *eps* locus (which encodes genes associated with exopolysaccharide biosynthesis) is located downstream of this island. The *eps* loci of *S. thermophilus* have previously been demonstrated to be genetically diverse and often harbour several transposase-encoding genes that are believed to contribute to the diversification of these clusters (63–65).

**Figure 5.**
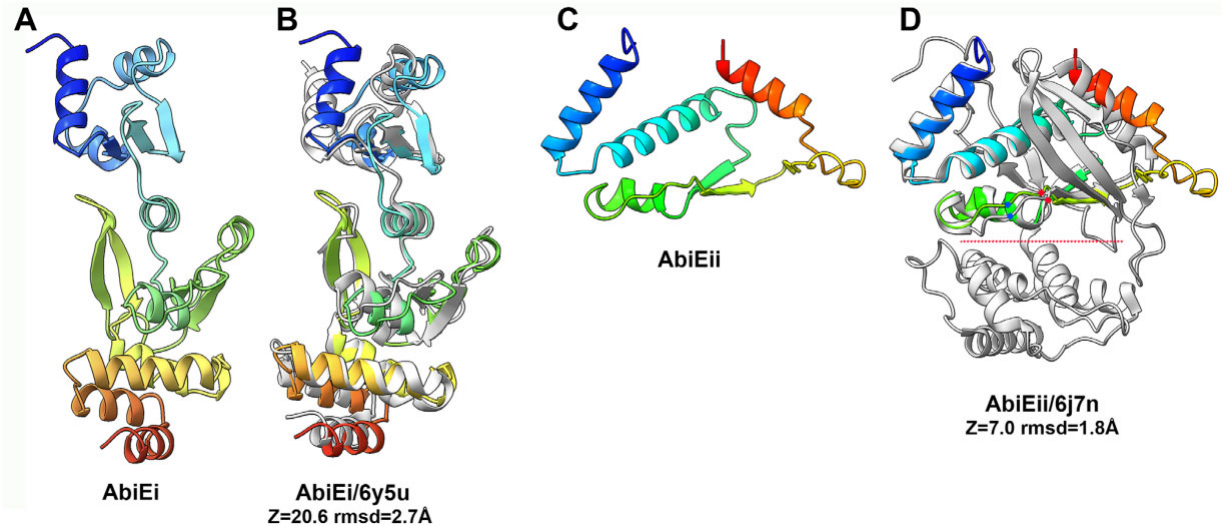
Structure prediction of the AbiE proteins. (**A**) Ribbon structure of the two domains AbiEi (rainbow colored). (**B**) Superposition of AbiEi (rainbow colored) with PDB 6y8q (grey), an antitoxin from *S. agalactiae* (*Z* = 20.6, rmsd = 2.7 Å). (**C**) Ribbon structure of AbiEii (rainbow colored). (**D**) Superposition of AbiEii (rainbow colored) with PDB 6j7n (grey), a guanylyltransferase-like toxin from *Mycobacterium tuberculosis* (*Z* = 7.0, rmsd = 1.8 Å). The red dot line indicates the catalytic crevice and the red and blue dots the positions of the catalytic residues in AbiEii and 6j7n.

The CR1-Defence Island is a highly conserved genomic region spanning approximately 21 kb (range: 20–22 kb) and represents the region which harbours CR1 (17 of 27 analysed genomes). CR1-defence Island is also associated with the presence of Type I or Type II R/M systems in some strains genomes. CR1-Defence Island is flanked by genes which encode septation and glutamine synthesis. This Defence Island incorporates approximately 20 genes, of which on average eight have no assigned function (Figure 1). Interestingly, in five (i.e. CNRZ887, CNRZ1151, 90730, 4052 and 4147 (Table 3, Figure 3A) of the ten strains that do not possess a CR1 region, the same relative genomic position is occupied by an AbiEi/Eii system, which is identical among these five strains (Figure 1). HHPred analysis established structural homology to toxin and antitoxin proteins (99% probability; PDB 6Y5U_A and 6Y8Q_B), while the 3D structure prediction of AbiEi was also found to superimpose well with the *S. agalactiae* antitoxin (PDB 6y5u) (Figure 5A and B). AlphaFold2 analysis of the product of ORF *90730_1387* revealed that the carboxy-terminus of the protein (AbiEii) is likely truncated since these toxins are bilobial enzymes with an active site located in a crevice between the two lobes encoded by the amino and carboxy-termini (Figure 5C and D). Besides, the 5-stranded β-sheet of 6j7n is also lacking. Therefore, while the catalytic residues of the active site are present (Figure 5D), the truncation of the protein is likely to affect the functionality of the system. This is consistent with plaque assay results where there is a limited impact on the plaquing efficiency of the phages tested in this study (Figure 3B).

### Strain-specific acquisitions enhance the anti-phage repertoire of *S. thermophilus*

While most of the anti-phage systems identified in this study are associated with the three CR-Defence Islands, genomes of individual strains were predicted to harbour unique anti-phage systems including SoFic, Hachiman, PD-T4-6 and Dodola. These systems can broadly be classified under the abortive infection category of phage resistance systems and their corresponding genes are positioned as standalone units at diverse locations across the genome. In functional assays, these systems do appear to elicit a minimal impact on phage resistance against the tested phages although it cannot be precluded that they operate synergistically with other systems in the host or that they are active against other phages beyond those evaluated in this study (Figure 3B) (66).

SoFic systems are reported to be widespread in bacterial genomes and a Class II Fic domain-encoding system was recently validated to be functional against coliphage T5 (15). These systems are typified by those with so-called ‘Fic’ domains with predicted protein AMPylase activity. Structural prediction of SoFic_MM20_ in the present study yielded a similar functional prediction (Supplementary Figures S3A, B and S5F).

PADLOC analysis identified two identical copies of a predicted SoFic-encoding gene in the genome of MM20 (Table 3). These genes occur at two disparate locations on the genome of MM20, i.e. *orf0248_MM20_* and *orf1073_MM20_*. This system is relatively rare among *S. thermophilus* strains with just three almost identical (98.55% sequence identity) homologues of this gene identified in the NCBI database. AlphaFold predicts SoFic as an α-helical protein (Supplementary Figures S3A and S5F). Dali reports hits of SoFic with a domain of 6i7g, an Adenosine Monophosphate-Protein Transferase Ficd with strong statistics (*Z* = 26.2; rmsd = 1.8) (Supplementary Figures S3B, C).

Hachiman was first identified in *Bacillus cereus* and incorporates two proteins termed HamB, which is a helicase and HamA, which is a nuclease. Hachiman has been reported to be present in 3.4% of microbial genomes evaluated (17). BLASTn analysis of *St19_0447* and *St19_0448* identified a single homologue of these genes in the *S. thermophilus* strain EU01 with 100% nucleotide sequence identity (E-value 0.00). AlphaFold2 predictions of HamA and HamB concur with previous functional studies. HamA is predicted as a compact α/β protein (Supplementary Figures S3D and S5C) and with hits in the Dali server against the EC869 toxin (4g6u), a Zn(2+)-dependent DNase (Supplementary Figures S3E). Superimposition of HamA on 4g6u yielded weak statistics (*Z* = 5.7; rmsd = 3.7Å). HamB is predicted as a multidomain α/β protein (Supplementary Figures S3F; S5D). Dali reports a strong hit for HamB with PDB 4u4c, an ATP-dependent RNA helicase (*Z* = 30.7; rmsd = 3.7 Å). So, Fic_MM20_ and Hachiman_ST19_ provide resistance against P738 (P738 group namesake) and SW16 (987 group member) (Figure 3B). It is noteworthy that members of the P738 and 987 groups of dairy streptococcal phages are relatively rarely encountered in dairy fermentations compared to members of the *Brussowvirus* and *Moineauvirus* genera. Therefore, we hypothesize that the consistent exposure of *Brussowvirus* and *Moineauvirus* members to dairy streptococcal strains and their phage-resistance systems has provided them an opportunity to overcome many of the identified phage-resistance systems through mutations or insertion/deletion events in contrast to members of the rare P738 and 987 streptococcal phage groups.

PD-T4-6 is one of several *E. coli* derived systems that is proposed to have DNA binding/cleavage activity although the specific mode of action remains to be elucidated (18). AlphaFold2 prediction of full-length PD-T4-6 is rather complex (Supplementary Figures S3H and S5E). The N-terminal region (residues 1–278; red box) displays a compact structure (Supplementary Figures S3H, I) and Dali server analysis of the N-terminal domain reports a clear hit (*Z* = 34.8; rmsd = 1.5 Å) with 4eqm (Supplementary Figures S3J), a serine/threonine protein kinase. This, and two subsequent modules, in tandem are formed of three antiparallel β-strands and one α-helix (Supplementary Figures S3H, K), a structure retrieved by Dali in PDB ID 3py9 (*Z* = 13, rmsd = 0.7 Å), a serine/threonine protein kinase binding module (Supplementary Figures S3L). Interestingly, the PD-T4-6 encoding gene is present in all analysed *S. thermophilus* genomes and was associated with a very mild phage-resistance phenotype in *Lactococcus* (Figure 3B).

Dodola is a two gene system (*dolA, dolB*) that has been shown to exert anti-phage activity against *Bacillus subtilis* phage SPP1 (15). Here, a single representative of this system was identified in the genome of strain 90729 (Table 3). DolA is of unknown function while DolB is predicted to possess a ClpB domain, which is typically associated with ATPase activity. The genes encoding Dodola_90729_ are located at the distal end of the genome of this strain and while it is not directly proximal to other phage-resistance systems, a Type II R/M system (GATC) is encoded by two genes that are located further downstream, which may be suggestive of a Defence Island. This is further supported by the variable occurrence of R/M systems in this genomic region in other strains. AlphaFold2 analysis of Dodola A (DolA) reports a three-domain protein: an α/β N-terminal domain, an α/β middle domain and an α-helical C-terminal domain (Supplementary Figures S3M). Dali reports hits of DolA with ATP-dependent Clp protease ATP-binding subunits 7xbk (Supplementary Figures S3N). To note, DolA is predicted with high confidence to assemble as a hexamer, as does 7xbk (Supplementary Figures S3O and S5I). DolB is predicted as a two domains protein associating a N-terminal α-helical domain to a α/β-C-terminal domain (Supplementary Figures S3P and S5H). Dali reports weak hits with several proteins involved in DNA/RNA binding/modification, which does not allow a precise functional assignment.

### Distribution of anti-phage systems in *S. thermophilus*

**Figure 6.**
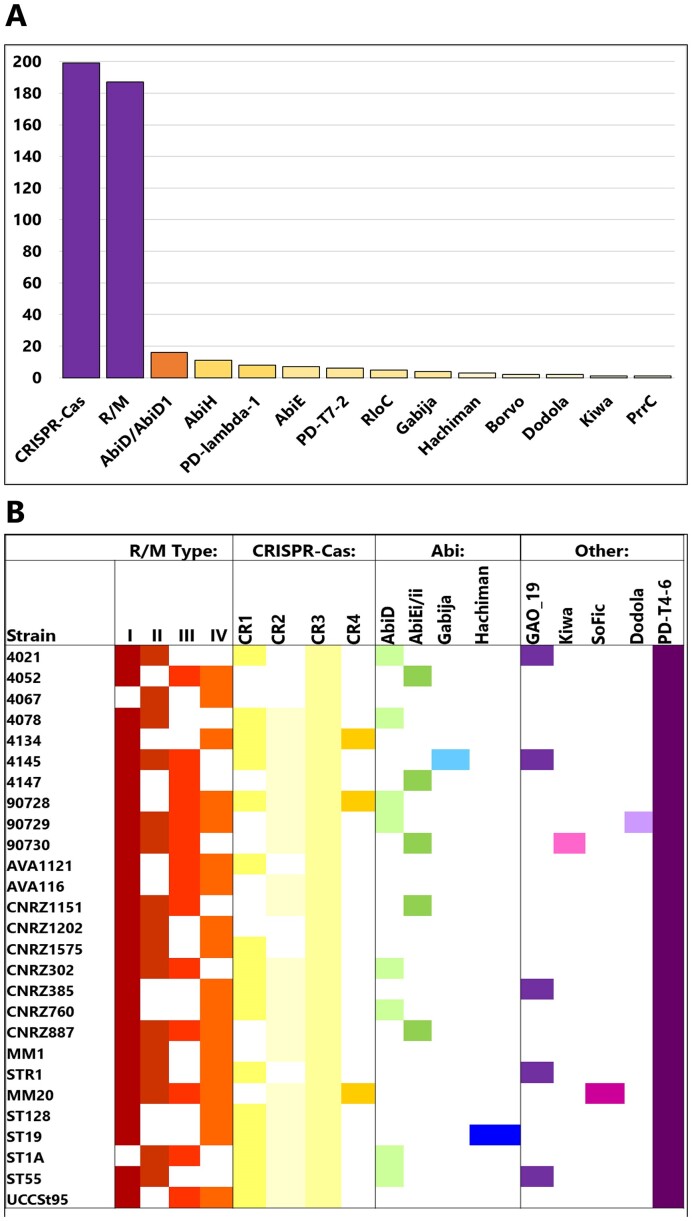
(**A**) Distribution of anti-phage systems identified in *S. thermophilus* in RefSeq database based on DefenseFinder analysis. The number of occurrences of the system in the RefSeq database of 462 entries is shown on the Y-axis and the systems are indicated on the X-axis. The bars are colour-coded to indicate the proportion of genomes in the RefSeq database with the respective antiphage system in the species where purple = 90–100; dark orange = >20; light orange = 5–19 and; pale yellow = <5. (**B**) Schematic depicting the phage-resistance landscape of the analysed 27 *S. thermophilus* strains’ genomes. The presence of systems is indicated by a coloured box and absence of the systems is indicated by a white box. Each system is coloured differently. The genomes of these strains harbour an average of seven anti-phage systems based on this analysis.

The presence and distribution of anti-phage systems in *S. thermophilus* genome sequence data available in the RefSeq database was analysed using DefenseFinder to (a) establish if the 27 genomes of our strain collection were reflective of the species overall and (b) to identify additional systems beyond those identified in the genomes sequenced as part of the present study. Using ‘Streptococcus thermophilus’ as a search term in the RefSeq database option of DefenseFinder, CRISPR-Cas and R/M systems were confirmed to be the globally dominant anti-phage systems in this species among 462 RefSeq entries (199 Cas and 187 R/M systems identified; Figure 6A). Beyond these dominant systems, a significantly smaller proportion of Abi systems including AbiD/D1, AbiH, AbiE and Gabija were also identified. Interestingly, while many systems are similar to those identified in the genomes of the 27 strains analysed in this study (Gabija, Kiwa, Dodola, AbiD, AbiE), there are a small number (and low number of occurrences) of additional predicted systems such as Borvo, PrrC, PD-lambda-1 and PD-T7-2. This highlights that while this species readily harnesses CRISPR-Cas and R/M systems, strain-specific acquisitions may confer an advantage on the hosting strain. As the number of genome sequences available within this species increases, it will be possible to establish the true extent of the diversification of the phage-resistome of dairy streptococci.

## Conclusions

In the present study, the phage resistance landscapes of 27 *S. thermophilus* strains were elucidated to establish the diversity and functionality of anti-phage systems in this species (Figure 6B). The innate and adaptive bacterial immune systems represented by R/M and CRISPR-Cas systems, respectively, are equipped to deal with ‘slow’-infecting phages or chronic infections (67). However, to cope with acute infections elicited by so-called ‘fast’-infecting phages, *S. thermophilus* has acquired abortive infection (Abi) systems and systems that are described to directly or indirectly activate abortive infection. These systems include AbiD, AbiE, SoFic, Dodola, Hachiman, Kiwa and GAO_19.

This collective of biological hurdles to deal with ‘slow’ and ‘fast’-infecting phages (or chronic and acute infections) provides *S. thermophilus* with a strain level and tailored series of defences to diminish the impact of phages present in their ecological niche. The strain level diversity of anti-phage systems (Figure 6B) may present a benefit at community level to ensure that *S. thermophilus* persists despite the pervasive presence of phages in dairy environments. Furthermore, since *S. thermophilus* has adapted to the dairy niche similar to *L. lactis/cremoris*, it is perhaps of broader benefit to the larger community of microorganisms that may be present in the same ecosystem. While receptor diversity/modification may act as the first line of defence against invading phages (25,68), the role of antiphage systems in limiting the impact on surrounding populations of cells is a critical component of maintaining community stability. Furthermore, in *Pseudomonas*, higher phage-resistance was correlated in strains whose genomes possessed a higher number of antiphage systems (69). Here, we identified nine distinct systems in addition to R/M and CRISPR-Cas systems that have not previously been functionally evaluated in *S. thermophilus*. Intriguingly, and in contrast to other bacterial genera, four of these systems (Hachiman, PD-T4-6, SoFic and Dodola) do not appear to be part of any obvious Defence Island. However, the identification of these ‘lone’ phage-resistance systems may form an anchor to identify additional phage-resistance systems and/or Defence Islands in this species in the future. In addition, several so-called ‘phage defence candidate’ (PDC) genes were identified in the genomes of these strains indicating that the potential reservoir of phage-resistance systems may be far greater than we currently realize.

Several of the identified systems were observed to be functional, not only in *S. thermophilus*, but also in *L. cremoris*. This cross-genus functionality provides strong evidence of the interactions of these lactic acid bacteria in agricultural and/or food production systems providing opportunities for mutually beneficial DNA transfer events. The current study reports the dedication of up to 2.5% of *S. thermophilus* genomes to anti-phage activity. It is tempting to suggest that this number represents the ‘tip of the iceberg’ and is likely significantly higher if we consider the newly identified systems in the present study forming the basis of much larger Defence Islands. Therefore, this study provides the foundation for systematic searches for phage-resistance systems within the identified Defence Islands and potential anchor regions for new Defence Islands in *S. thermophilus*.

## Supplementary Material

gkae814_Supplemental_File

## Data Availability

All coordinates of predicted structures are deposited in the open data repository Zenodo (DOI 10.5281/zenodo.11221718). All RM-associated methylome and functionality data is available through REBASE (http://rebase.neb.com/rebase/).
