# Supplementary material for: Phage defence loci of Streptococcus thermophilus—tip of the anti-phage iceberg?
Source: Nucleic Acids Res. 2024 Sep 24;52(19):11853–69. doi: 10.1093/nar/gkae814 (PMC11514479; doi:10.1093/nar/gkae814)
Supplement: gkae814_Supplemental_File [file gkae814_supplemental_file.pdf]

Supplementary Figure S1:

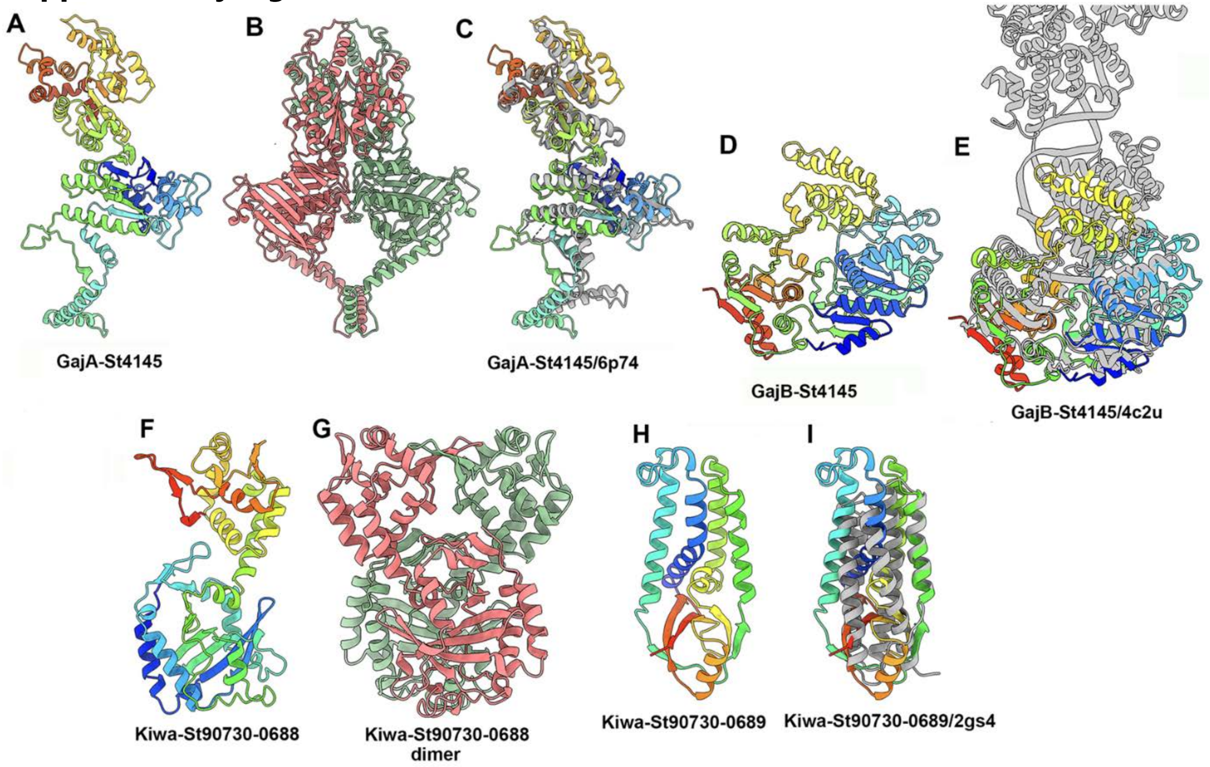

Fig. S1 Structure prediction of Gabija and Kiwa proteins**. (A)** Ribbon structure of $GajA_{4145}$ (rainbow coloured); **(B)** Dimer of $GajA_{4145}$. **(C)** Superimposition of $GajA_{4145}$ with PDB 6p74, a Class 1 OLD family nuclease (grey; Z=20.2, rmsd =5.6 Å). (D) Ribbon structure of $GajB_{4021}$ (rainbow coloured); **(E)** Superimposition of $GajB_{4021}$ with PDB 4c2u, a helicase in complex with DNA (grey; Z=24.1, rmsd =3.7 Å). **(F)** Ribbon structure of $KwaB_{90730}$ (rainbow coloured); **(G)** Ribbon structure of $KwaB_{90730}$ dimer. **(H)** Ribbon structure of $KwaA_{90730}$ (rainbow coloured); **(I)** Superimposition of $KwaA_{90730}$ with PDB 2gs4 (Z=5.1, rmsd =5.7 Å).

Supplementary Figure S2:

Fig. S2. AlphaFold prediction of AbiD proteins encoded by *S. thermophilus* **(A)** ST55 (orange), **(B)** 4078 (blue), **(C)** 4021 (pink) and **(D)** *Lactococcus* (green). The lower panel **(E)** is a superimposition of AbiD-ST55 with PDB ID 6iv9 (Z=7.7; rmsd=3.9Å) a CRISPR-associated endoribonuclease Cas13D. **(F)** Superimposition of the four AbiD proteins showing their common central core Cas13D-like fold.

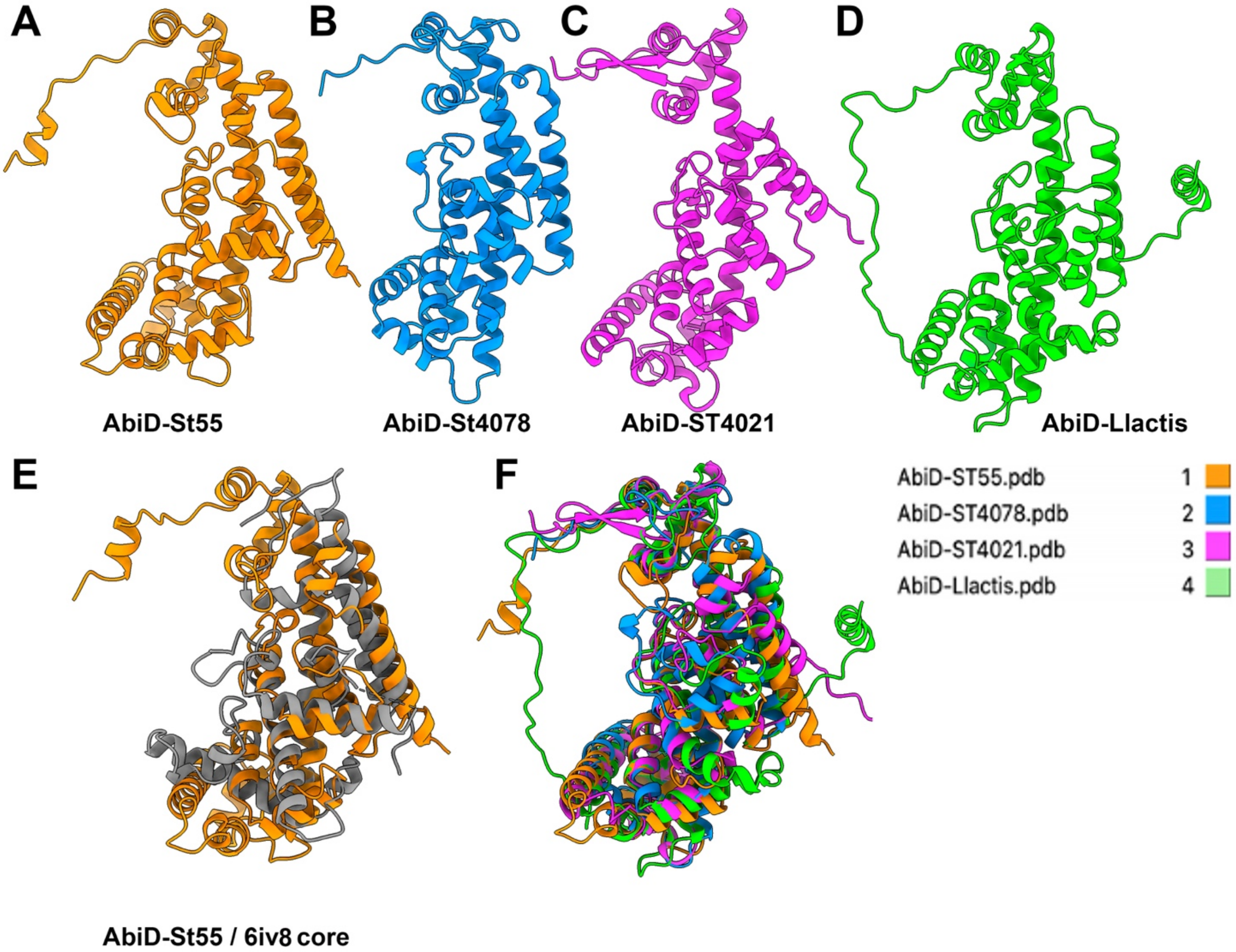

Supplementary Figure S3:

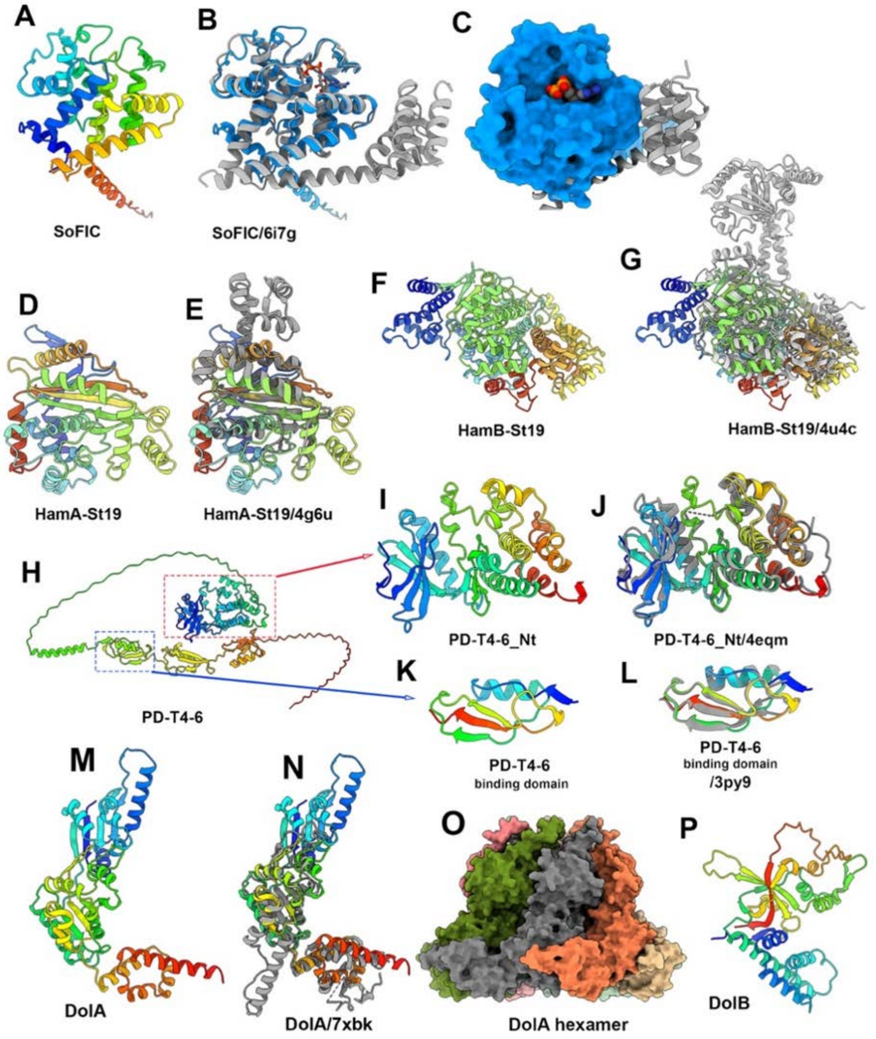

Fig. S3. Structure predictions of Sofic, Hachiman, PD-T4-6and Dodola. **(A)** Ribbon structure of SoFIC (rainbow coloured); **(B)** Superimposition of SoFIC with PDB 6i7g (grey; Z=26.2, rmsd =1.8 Å); **(C)** Molecular surface of SoFIC with the sphere model of 6iTg ATP inside its catalytic crevice. **(D)** Ribbon structure of HamA$_{ST19}$ (rainbow coloured); **(E)** Superimposition of HamA$_{ST19}$ with PDB 4g6u (grey; Z=5.5, rmsd =5.3 Å), the EC869 toxin; **(F)** Ribbon structure of HamB$_{ST19}$ (rainbow coloured); **(G)** Superimposition of HamB$_{ST19}$ with PDB 4u4c (grey; Z=30.4, rmsd =3.7 Å); **(H)** Ribbon structure of full-length PD-T4-6 (rainbow coloured); **(I)** N-terminal domain of PD-T4-6; **(J)** Superimposition of PD-T4-6-Nt with 4eqm (grey), a serine/threonine protein kinase; **(K)** structure of one of the three C-terminal domains; **(L)** Superimposition of one domain with 3py9, a binding domain; **(M)** Ribbon structure of DolA (rainbow coloured); **(N)** Superimposition of DolA with PDB 7xbk (grey; Z=21.2, rmsd =2.9 Å); **(O)** Side-view of the molecular surface of the DolA hexamer; **(P)** Ribbon structure of DolB (rainbow coloured).

Supplementary Figure S4:

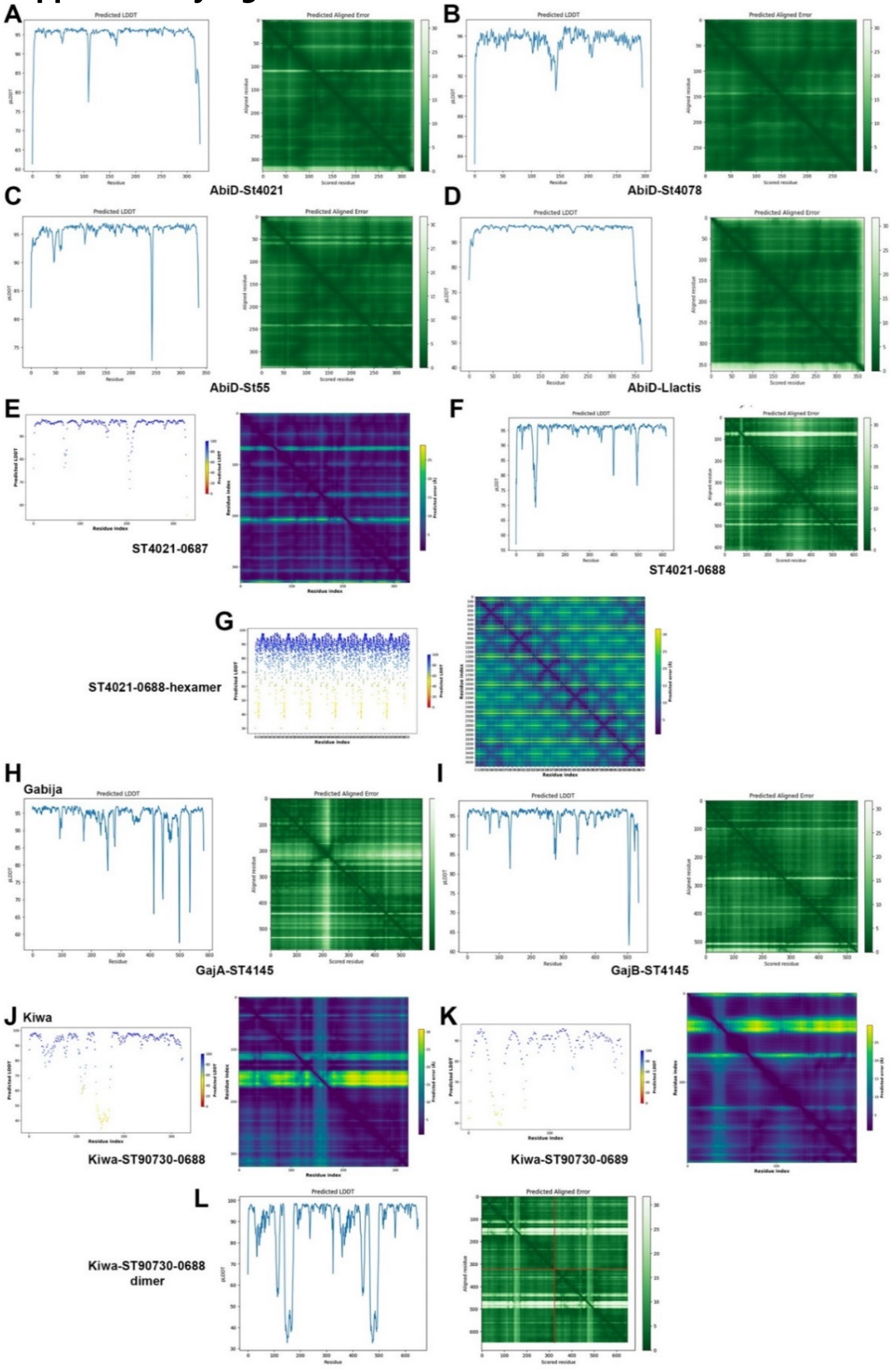

Fig. S4. pLDDT values and predicted aligned errors (PAE) plots of predicted structures (Part 1)

The pLDDT plots (left) and PAE plots (right) correspond to unique proteins or complexes. The pLDDT values (Y axis) are plotted for each residue (X axis). In PAE plots (residue number *vs* residue number) of complexes, the folded proteins or domains form squares along the diagonal while interactions between proteins or domains appear as squares at the vertical/horizontal intersection of the domains/proteins.

Supplementary Figure 5: pLDDT values and predicted aligned errors (PAE) plots of predicted structures (Part 2)

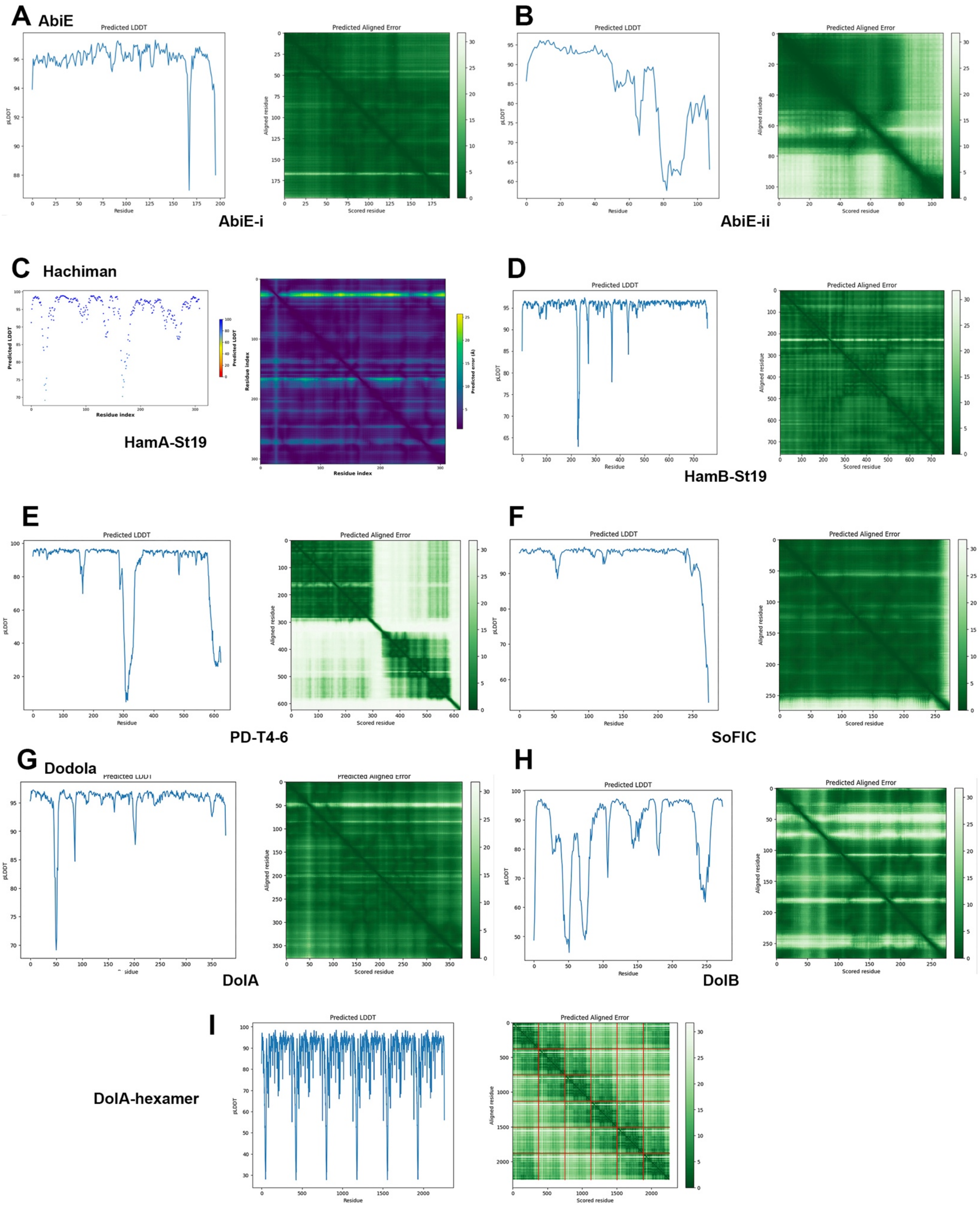

Supplementary Table S1. Oligonucleotides used in this study. These oligonucleotides were used to clone genes into pNZ44 and pPTPi and for PCR-based screening

Oligonucleotide name	Oligonucleotide sequence (5′ → 3′)
**pPTPi[Ψ] constructs**	
**4078_RM_FW**	AGCAGCGTCGACAGGAGGCACTCACCATGGTTTCTAGAAGAGAGTTTT
**4078_RM_RV**	AGCAGCGCATGCCTACTCCACCTTCACCTG
**ST1A_RM_FW**	AGCAGCGGATCCAGGAGGCACTCACCATGGCAAAAATTGAACCGAAAA
**ST1A_RM_RV**	AGCAGCGCATGCTCACTCATCCTCCTCGATT
**MM1_RM_FW**	AGCAGCGGATCCAGGAGGCACTCACCATGAACGCTAGAAAACAGAAAA
**MM1_RM_RV**	AGCAGCGAATTCTTATTTAGCTAGAGCTTCATACA
**pNZ44[Ψ] constructs**	
**PDT46_4078_FW**	AAAAAACCATGGATGATCCAAGTTGGCAAATTG
**PDT46_4078_RV**	AAAAAAGAGCTCTTATTGCTCTGTGTGAGTAG
**PDT46_4078_U_FW**	AAAAAACCATGGGTTAACAGTCCTGGTCTTGT
**PDT46_4078_U_RV**	AAAAAAGAGCTCCGCGTTCATCATAAAAGAATCT
**Sofic_MM20_FW**	AAAAAACCATGGATGGATTTAATTACAAGACTTAAAG
**Sofic_MM20_RV**	AAAAAAGAGCTCTTATAACGAAAAACCTTTTGTTTTG
**Dodola_90729_FW**	AAAAAAGGTACCTTGGCGGAAAATTTAAAAAAAAATA
**Dodola_90729_RV**	AAAAAAGAGCTCTTAGTTATGATTTTTATCGATTCTA
**AbiEi_90730_FW**	AAAAAACCATGGCGAAAGGATAGTGCTATTATGAA
**AbiEi_90730_RV**	AAAAAAGAGCTCTCCTAATCTCTCAATTCTTCAAG
**AbiEi_90730_U_FW**	AAAAAACCATGGTCTAGAAGAGTTGGATGAAATG
**AbiEi_90730_U_RV**	AAAAAAGAGCTCGGTTGGATTCAGTAAAGAACAA
**Kiwa_90730_FW**	AAAAAACCATGGGGAAAGAGGCATAAATTGATTC
**Kiwa_90730_RV**	AAAAAAGAGCTCTGTTACCTATTTTCTTTTTAAAATG
**GAO_R1_FW**	AAAAAACCATGGGTATTTTTAGGAGTTAATTATGGAA
**GAO_R1_RV**	AAAAAAGAGCTCGTCTGGTTTCATTTGCCTTC
**Hach_ST19_FW**	AAAAAACCATGGGAAAAGGAAAAGTAATATTGAGAA
**Hach_ST19_RV**	AAAAAAGAGCTCCCCAGTCTTATCTTTTATTCAA
**AbiD_ST55_FW**	AAAAAACCATGGAGGAGAGGTTACTGAATATTTTAA
**AbiD_ST55_RV**	AAAAAAGAGCTCCCATTAATTTCGTTTTTCGTCAAT
**AbiD_ST55_U_FW**	AAAAAACCATGGGGATTTGTTAGCATATTGTTTTG
**AbiD_ST55_U_RV**	AAAAAAGAGCTCAGGAGGACTGACGCATCA
**GAO_4021_FW**	AAAAAACCATGGCTTGAGGAATATACATATGGTTG
**GAO_4021_RV**	AAAAAAGAGCTCTCAACTCCACAATTCAGTCAAA

**AbiD_4021_FW**	AAAAAACCATGGTGTTAAGTGAGAATACTATGATTG
**AbiD_4021_RV**	AAAAAAGAGCTCTTATCTAGTTCTTCTGCGTTTC
**AbiD_4021_U_FW**	AAAAAACCATGGCGTGTGGTGTAAGAATGTTG
**AbiD_4021_U_RV**	AAAAAAGAGCTCCAGGCAAGAGACTAGCAC
**AbiD_4078_FW**	AAAAAACCATGGAAGGGATTGCCATTATGCAAC
**AbiD_4078_RV**	AAAAAAGAGCTCCTATACGTTGTCAACTATTTTTTG
**AbiD_4078_U_FW**	AAAAAACCATGGGATGACAAAGCGATTTTTGAG
**AbiD_4078_U_RV**	AAAAAAGAGCTCCGAAATGATATAGAAAGAGTCG
**Gabija_4145_FW**	AAAAAACCATGGATTGGATAGGATAACTAATGAAAT
**Gabija_4145_RV**	AAAAAAGAGCTCCCTTAGTTGTATAGGCATTATTA
**Gabija_4145_U_FW**	AAAAAACCATGGGGACAGGTGAAGGTATAAAAT
**Gabija_4145_U_RV**	AAAAAAGAGCTCACCTCCTTGATAATTCAGAAC
**Mutant construction**	
**034RM_1**	GAATTCCTGCAGCCCACTTGGATACTCGGTAAGAG
**034RM_2**	CTAATGTACCTCATTTCCATAGTTGCC
**034RM_3**	TGGAAATGAGGTACATTAGAAGCTGGAGACG
**034RM_4**	ACTAGTGGATCCCCCAATCACCAAACGTTGGTCTG
**Screening/Sequencing primers**	
**pPTPi_FW**	TGATTTCGTTCGAAGGAACTA
**pPTPi_RV**	TGGCGGACAATAAGTCCTC
**pNZ44_FW**	CAATTGTAACCCATCCAGGA
**pNZ44_RV**	TGGCTATCAATCAAAGCAACA
**4078_RM_Seq_1**	TCACTCTATCAAAAGGAAAGAC
**4078_RM_Seq_2**	GCTCATCCACGTTATGTCTCAC
**4078_RM_Seq_3**	ATCGACGAAGTGAGGGATAGAC
**4078_RM_Seq_4**	TCTTTACCCATGCTGGTCTAGG
**4078_RM_Seq_5**	CTATCATCGCTAATCGCCATCC
**4078_RM_Seq_6**	GCATTGGAGCAGGAGATTGAAG
**4078_RM_Seq_7**	TCCCAGACCAGAATGCCAAACC
**ST1A_RM_Seq_1**	CCGCGAGTTACTTACTGATG
**ST1A_RM_Seq_2**	TGGAACTTCGTCGGTGAAAC
**ST1A_RM_Seq_3**	CGTCCGTGACTTAATCTCTG
**ST1A_RM_Seq_4**	TGCCCGTGATGATAAGGAAC
**ST1A_RM_Seq_5**	CGTGGTGCAAGGTATTATCC
**ST1A_RM_Seq_6**	CACGGCAAACCTCTTGAATG

**MM1_RM_Seq_1**	CAATGGTGGGATGTTCTCTG
**MM1_RM_Seq_2**	GCAAGGAGAACAGGCAAGTC
**MM1_RM_Seq_3**	AAGACCGATGGACGTTATGG
**GAO_R1_1**	CATGGTTCGATTAACTGGACTTC
**GAO_R1_2**	GGAACTATCCAGAATAAGTATGA
**Hach_ST19_1**	TGCTTTTACCAGTTGGTGATTCT
**Hach_ST19_2**	TCAAAAACCAGAAACTAGAGTAA
**GAO_4021_1**	ATCCAAGAAATCTGAATGTCATTA
**GAO_4021_2**	GATAAACTTCCAATAACTTCAGA
**Gabija_4145_1**	CGAGGAGCCAGAAAACAGTC
**Gabija_4145_2**	AGTGATCAGAAAATTAGGAATGA
**pGH9L**	TGGTACAGCTGAAGTTGTTG
**pGH9R**	GTATACTACTGACAGCTTCC
**034RMdelL**	CTCCAAGAACTTTGCTTCTG
**034RMdelR**	CCATTGTGTCGCTTGTATTG

Ψ Introduced restriction enzyme sites are singly underlined, Shine-Dalgarno sequences are doubly underlined.

Supplementary Table S2. Details of plasmids associated with analysed strains

***S. thermophilus* strain**	**Plasmid name**	**Size (kb)**	**# predicted ORFs**	**Identified phage-resistance systems**
ST1A	p1AA	3.529	2	
4021	p4021A	3.529	2	
4067	p4067A	3.385	3	
4147	p4147A	5.802	4	
AVA116	pAVA116A	5.430	5	
CNRZ1202	pCNRZ1202A	7.493	7	Type II R/M
	pCNRZ1202B	5.728	5	
ST55	pST55A	5.175	5	
ST128	pST128A	8.145	6	

Supplementary Table S3: Type I Active Systems (32)

Organism	Enzyme	Rec seq (meth base/type)
Streptococcus thermophilus 4021	M.Sth4021I	GCANNNNNNNTCG
Streptococcus thermophilus 4052	M.Sth4052I	CGANNNNNNTGA
Streptococcus thermophilus 4078	M.Sth4078I	CCAYNNNNNTGA
Streptococcus thermophilus 4134	M.Sth4134I	TGAANNNNNNNTYTC
	M.Sth4134II	GCANNNNNNCTAR
Streptococcus thermophilus 4145	M.Sth4145I	GGANNNNNRTTG
Streptococcus thermophilus 4147	M.Sth4147I	GAAYNNNNNNTAYG
Streptococcus thermophilus 90728	M.Sth90728I	YTCANNNNNNTGC
Streptococcus thermophilus 90729	M.Sth90729I	GGANNNNNNNTCG
Streptococcus thermophilus 90730	M.Sth90730I	CGANNNNNNTAC
Streptococcus thermophilus AVA1121	M.Sth1121I	GNAAYNNNNNNNTAYC
	M.Sth1121II	CAACNNNNNTCA
Streptococcus thermophilus AVA116	M.Sth116I	GAAYNNNNNCTAR
	M.Sth116III	CCANNNNNNNTCG
Streptococcus thermophilus CNRZ1202	M.Sth1202I	CACNNNNNRTGA
Streptococcus thermophilus CNRZ1575	M.Sth1575I	ACGNNNNNNNTAYC
	M.Sth1575II	GGANNNNNNTGG
Streptococcus thermophilus CNRZ302	M.SthZ302I	GGANNNNNNTGC
Streptococcus thermophilus CNRZ385	M.Sth385I	TACBNNNNNNTGC
Streptococcus thermophilus CNRZ760	M.Sth760I	GACNNNNNTGC
	M.Sth760II	CRTANNNNNNAGT
Streptococcus thermophilus CNRZ887	M.Sth887I	CRTANNNNNATC
Streptococcus thermophilus MM1	M.SthMM1I	GGANNNNNNTCC
Streptococcus thermophilus MM20	M.SthMM20I	GGANNNNNNTAC
Streptococcus thermophilus R1	M.SthR1II	GATTNNNNNNTCG
Streptococcus thermophilus ST128	M.Sth128I	ACGNNNNNNNTAYC
	M.Sth128II	GGANNNNNNTAC
Streptococcus thermophilus ST19	M.Sth19I	GGANNNNNNNTAAT
	M.Sth19II	TGAANNNNNNNVTTG
Streptococcus thermophilus ST55	M.Sth55I	TCTANNNNNNNTAGY
Streptococcus thermophilus strain 1A	M.Sth1AI	CRTANNNNNNTGG
Streptococcus thermophilus UCCSt95	M.Sth95I	GGANNNNNNTGG

Supplementary Table S4: Type II Active Systems (11)

Organism	Enzyme	Rec seq (meth base/type)
Streptococcus thermophilus 90730	Sth90730II	CAGAG
Streptococcus thermophilus CNRZ1151	Sth1151I	CAGAG
Streptococcus thermophilus CNRZ1202	M.Sth1202II	GATC
Streptococcus thermophilus CNRZ887	M.Sth887II	CAGAG
	Sth887II	CAGAG
Streptococcus thermophilus MM1	SthMM1II	CAGRAG
Streptococcus thermophilus MM20	M.SthMM20III	GAAGT
	SthMM20III	GAAGT m6A
Streptococcus thermophilus R1	M.SthR1I	GAAGT
	SthR1I	GAAGT
Streptococcus thermophilus ST55	Sth55II	CAGRAG

Supplementary Table S5: Type III active Systems (6)

Organism	Enzyme	Rec seq (meth base/type)
Streptococcus thermophilus AVA116	M.Sth116II	CGANAG
Streptococcus thermophilus CNRZ302	M.SthZ302II	CGCAT
Streptococcus thermophilus MM20	M.SthMM20II	CGCAT
Streptococcus thermophilus strain 1A	M.Sth1AII	CGWAAT
Streptococcus thermophilus UCCSt95	M.Sth95II	CGCAT
	M.Sth95III	ACAGC

Supplementary Table S6: Type I Inactive Systems (46)

Organism	Enzyme	Rec seq (meth base/type)
Streptococcus thermophilus 4052	M.Sth4052ORF4065P	- m6A
Streptococcus thermophilus 4067	M1.Sth4067ORF4200P	- m6A
	M2.Sth4067ORF4200P	- m6A
Streptococcus thermophilus 4134	M1.Sth4134ORF4310P	- m6A
	M2.Sth4134ORF4310P	- m6A
Streptococcus thermophilus 4145	M1.Sth4145ORF4170P	- m6A
	M2.Sth4145ORF4170P	- m6A
Streptococcus thermophilus 4147	M1.Sth4147ORF4210P	- m6A
	M2.Sth4147ORF4210P	- m6A

Streptococcus thermophilus 90728	M1.Sth90728ORF4395P	- m6A
	M2.Sth90728ORF4395P	- m6A
Streptococcus thermophilus 90729	M1.Sth90729ORF4435P	- m6A
	M2.Sth90729ORF4435P	- m6A
Streptococcus thermophilus 90730	M1.Sth90730ORF4170P	- m6A
	M2.Sth90730ORF4170P	- m6A
Streptococcus thermophilus AVA1121	M1.Sth1121ORF4175P	- m6A
	M2.Sth1121ORF4175P	- m6A
Streptococcus thermophilus AVA116	M1.Sth116ORF4030P	- m6A
	M2.Sth116ORF4030P	- m6A
Streptococcus thermophilus CNRZ1151	M.Sth1151ORF3645P	GNNTAYNNNNNNTGC m6A
	M1.Sth1151ORF4190P	- m6A
	M2.Sth1151ORF4190P	- m6A
Streptococcus thermophilus CNRZ1202	M1.Sth1202ORF4140P	- m6A
	M2.Sth1202ORF4140P	- m6A
Streptococcus thermophilus CNRZ1575	M1.Sth1575ORF4310P	- m6A
	M2.Sth1575ORF4310P	- m6A
Streptococcus thermophilus CNRZ302	M1.SthZ302ORF4280P	- m6A
	M2.SthZ302ORF4280P	- m6A
Streptococcus thermophilus CNRZ385	M.Sth385ORF4285P	- m6A
Streptococcus thermophilus CNRZ760	M1.Sth760ORF4425P	- m6A
	M2.Sth760ORF4425P	- m6A
Streptococcus thermophilus CNRZ887	M1.Sth887ORF4110P	- m6A
	M2.Sth887ORF4110P	- m6A
Streptococcus thermophilus MM1	M.SthMM1ORF5195P	- m6A
Streptococcus thermophilus MM20	M.SthMM20ORF5900P	- m6A
Streptococcus thermophilus R1	M1.SthR1ORF4325P	- m6A
	M2.SthR1ORF4325P	- m6A
Streptococcus thermophilus ST128	M1.Sth128ORF4290P	- m6A
	M2.Sth128ORF4290P	- m6A
Streptococcus thermophilus ST19	M.Sth19ORF4280P	- m6A
Streptococcus thermophilus ST55	M1.Sth55ORF4365P	- m6A
	M2.Sth55ORF4365P	- m6A
Streptococcus thermophilus strain 1A	M1.Sth1AORF4285P	- m6A
	M2.Sth1AORF4285P	- m6A
Streptococcus thermophilus UCCSt95	M1.Sth95ORF4165P	- m6A
	M2.Sth95ORF4165P	- m6A

Supplementary Table S7: Type II Inactive Systems (49)

**Organism**	**Enzyme**	**Rec seq (meth base/type)**
Streptococcus thermophilus 4021	M.Sth4021ORF3410P	-
	M.Sth4021ORF3535P	GCSGC m5C

Streptococcus thermophilus 4052	M.Sth4052ORF3335P	-
Streptococcus thermophilus 4067	M.Sth4067ORF3440P	-
	M.Sth4067ORF7395P	GATC m5C
Streptococcus thermophilus 4078	M.Sth4078ORF3405P	-
	M.Sth4078ORF3530P	GATC m5C
	M.Sth4078ORF9015P	- m5C
Streptococcus thermophilus 4145	M.Sth4145ORF3315P	-
	M.Sth4145ORF3445P	GATC m5C
	M.Sth4145ORF8840P	- m5C
Streptococcus thermophilus 4147	M.Sth4147ORF3375P	-
Streptococcus thermophilus 90728	M.Sth90728ORF3310P	- m5C
	M.Sth90728ORF3570P	-
Streptococcus thermophilus 90729	M.Sth90729ORF3330P	- m5C
	M.Sth90729ORF3590P	-
	M.Sth90729ORF8980P	GATC m5C
Streptococcus thermophilus 90730	M.Sth90730ORF3335P	-
	M.Sth90730ORF6370P	CAGAG
Streptococcus thermophilus AVA1121	M.Sth1121ORF3350P	-
Streptococcus thermophilus AVA116	M.Sth116ORF3260P	-
Streptococcus thermophilus CNRZ1151	M.Sth1151ORF3340P	-
	M.Sth1151ORF6445P	GAAGT
Streptococcus thermophilus CNRZ1202	M.Sth1202ORF3400P	-
Streptococcus thermophilus CNRZ1575	M.Sth1575ORF3505P	-
	Sth1575ORF2800P	GCAAAT m6A
Streptococcus thermophilus CNRZ302	M.SthZ302ORF3430P	-
	M.SthZ302ORF3555P	GATC m5C
	M.SthZ302ORF3925P	- m5C
	M.SthZ302ORF9005P	- m5C
Streptococcus thermophilus CNRZ385	M.Sth385ORF3460P	-
	M1.Sth385ORF9165P	- m5C
	M2.Sth385ORF9165P	- m5C
Streptococcus thermophilus CNRZ760	M.Sth760ORF3355P	- m5C
	M.Sth760ORF3665P	-
	M.Sth760ORF7710P	- m5C
Streptococcus thermophilus CNRZ887	M.Sth887ORF3325P	-
Streptococcus thermophilus MM20	M.SthMM20ORF6685P	-
	SthMM20ORF7340P	GCAAAT m6A
Streptococcus thermophilus R1	M.SthR1ORF3345P	-
Streptococcus thermophilus ST128	M.Sth128ORF3545P	-
Streptococcus thermophilus ST19	M.Sth19ORF2475P	- m5C
	M.Sth19ORF3425P	-
Streptococcus thermophilus ST55	M.Sth55ORF3310P	- m5C
	M.Sth55ORF3590P	-
	M.Sth55ORF3715P	- m5C
Streptococcus thermophilus strain 1A	M.Sth1AORF3395P	-
	M.Sth1AORF3520P	GCSGC m5C
Streptococcus thermophilus UCCSt95	M.Sth95ORF3370P	-

Supplementary Table S8: M5C methylase genes (20)

Organism	Enzyme	Recognition Sequence
Streptococcus thermophilus 4021	M.Sth4021ORF3535P	GCSGC
Streptococcus thermophilus 4067	M.Sth4067ORF7395P	GATC
Streptococcus thermophilus 4078	M.Sth4078ORF3530P	GATC
	M.Sth4078ORF9015P	-
Streptococcus thermophilus 4145	M.Sth4145ORF3445P	GATC
	M.Sth4145ORF8840P	-
Streptococcus thermophilus 90728	M.Sth90728ORF3310P	-
Streptococcus thermophilus 90729	M.Sth90729ORF3330P	-
	M.Sth90729ORF8980P	GATC
Streptococcus thermophilus CNRZ302	M.SthZ302ORF3555P	GATC
	M.SthZ302ORF3925P	-
	M.SthZ302ORF9005P	-
Streptococcus thermophilus CNRZ385	M1.Sth385ORF9165P	-
	M2.Sth385ORF9165P	-
Streptococcus thermophilus CNRZ760	M.Sth760ORF3355P	-
	M.Sth760ORF7710P	-
Streptococcus thermophilus ST19	M.Sth19ORF2475P	-
Streptococcus thermophilus ST55	M.Sth55ORF3310P	-
	M.Sth55ORF3715P	-
Streptococcus thermophilus strain 1A	M.Sth1AORF3520P	GCSGC

Supplementary Table S9: Type III Inactive Systems (8)

Organism	Enzyme	Rec seq (meth base/type)
Streptococcus thermophilus 4052	M.Sth4052ORF4190P	CGCAT m6A
Streptococcus thermophilus 4145	M.Sth4145ORF4280P	CGWAAT m6A
Streptococcus thermophilus 4147	M.Sth4147ORF4330P	CGCAT m6A
Streptococcus thermophilus 90728	M.Sth90728ORF4495P	CGCAT m6A
Streptococcus thermophilus 90729	M.Sth90729ORF4565P	CGCAT m6A

Streptococcus thermophilus 90730	M.Sth90730ORF4290P	CGCAT m6A
Streptococcus thermophilus CNRZ1151	M.Sth1151ORF4310P	CGCAT m6A
Streptococcus thermophilus CNRZ887	M.Sth887ORF4230P	CGCAT m6A

Supplementary Table S10: Type IV Systems (Unknown activity) (18)

Organism	Enzyme	Rec seq (meth base/type)
Streptococcus thermophilus 4052	Sth4052ORF3465P	-
Streptococcus thermophilus 4067	Sth4067ORF3570P	-
Streptococcus thermophilus 4134	Sth4134ORF3555P	-
Streptococcus thermophilus 90728	Sth90728ORF3700P	-
Streptococcus thermophilus 90729	Sth90729ORF3720P	-
Streptococcus thermophilus AVA1121	Sth1121ORF3480P	-
Streptococcus thermophilus AVA116	Sth116ORF3390P	-
Streptococcus thermophilus CNRZ1202	Sth1202ORF3540P	-
Streptococcus thermophilus CNRZ1575	Sth1575ORF3640P	-
Streptococcus thermophilus CNRZ385	Sth385ORF3595P	-
Streptococcus thermophilus CNRZ760	Sth760ORF3800P	-
Streptococcus thermophilus CNRZ887	Sth887ORF3455P	-
Streptococcus thermophilus MM1	SthMM1ORF3610P	-
Streptococcus thermophilus MM20	SthMM20ORF6555P	-
Streptococcus thermophilus R1	SthR1ORF3475P	-
Streptococcus thermophilus ST128	Sth128ORF3675P	-
Streptococcus thermophilus ST19	Sth19ORF3555P	-
Streptococcus thermophilus UCCSt95	Sth95ORF3505P	-

Supplementary Table S11: Systems recognizing CGCAT (10)

Organism	Enzyme	Rec seq (meth base/type)
Streptococcus thermophilus 4052	M.Sth4052ORF4190P	CGCAT m6A
Streptococcus thermophilus 4147	M.Sth4147ORF4330P	CGCAT m6A
Streptococcus thermophilus 90728	M.Sth90728ORF4495P	CGCAT m6A
Streptococcus thermophilus 90729	M.Sth90729ORF4565P	CGCAT m6A
Streptococcus thermophilus 90730	M.Sth90730ORF4290P	CGCAT m6A
Streptococcus thermophilus CNRZ1151	M.Sth1151ORF4310P	CGCAT m6A
Streptococcus thermophilus CNRZ302	M.SthZ302II	CGCAT
Streptococcus thermophilus CNRZ887	M.Sth887ORF4230P	CGCAT m6A
Streptococcus thermophilus MM20	M.SthMM20II	CGCAT
Streptococcus thermophilus UCCSt95	M.Sth95II	CGCAT

Supplementary Table S12. Efficiency of plaquing (EOP) of lactococcal phages in the presence or absence of Type I, Type II or Type III R/M systems

**VES7862 pPTPi derivative**	**sk1**	**c2**	**p2**	**jj50**	**712**	**62606**
Empty vector control	1	1	1	1	1	1
4078_RM I Uninduced	1.07 ± 0.12	1.02 ± 0.05	1.14 ± 0.06	1.11 ± 0.12	0.96 ± 0.08	1.10 ± 0.11
4078_RM I Induced	4.0×10^{-5} ± 1.1×10^{-5}	6.8×10^{-4} ± 1.2×10^{-4}	2.6×10^{-5} ± 9.5×10^{-6}	5.1×10^{-5} ± 5.3×10^{-6}	5.9×10^{-4} ± 1.7×10^{-4}	1.0×10^{-3} ± 2.9×10^{-4}
MM1_RM II Uninduced	1.11 ± 0.20	0.92 ± 0.10	1.27 ± 0.19	1.11 ± 0.11	0.92 ± 0.08	0.92 ± 0.10
MM1_RM II Induced	2.1×10^{-4} ± 5.5×10^{-5}	6.1×10^{-3} ± 5.9×10^{-4}	1.1×10^{-4} ± 3.1×10^{-5}	5.4×10^{-4} ± 1.6×10^{-4}	9.7×10^{-4} ± 1.8×10^{-4}	1.6×10^{-3} ± 4.1×10^{-4}
ST95_RM III Uninduced	1.06 ± 0.09	0.99 ± 0.07	1.03 ± 0.07	1.11 ± 0.12	0.97 ± 0.11	0.84 ± 0.12
ST95_RM III Induced	1.1×10^{-4} ± 3.1×10^{-5}	9.4×10^{-4} ± 7.8×10^{-5}	1.6×10^{-5} ± 4.8×10^{-6}	3.8×10^{-5} ± 1.1×10^{-5}	6.3×10^{-4} ± 6.7×10^{-5}	3.2×10^{-4} ± 8.4×10^{-5}

Supplementary Table S13. Efficiency of plaquing of representative *S. thermophilus* and *L. cremoris* phages on strains harbouring constructs expressing predicted phage-resistance systems. Phages SW13 (*Brussowvirus*), STP1 (*Moineauvirus*), SW16 (987 group), P738 (P738 group), sk1 (*Skunavirus*) and c2 (*Ceduovirus*) were used in these assays. Blue boxes represent those where a reduction in plaque size was observed. Presented data are the average of (at least) triplicate assays.

**Predicted anti-phage system_source strain**	**Plaquing efficiency of: *S. thermophilus* phages:**				***L. cremoris* phages:**	
	**SW13**	**STP1**	**P738**	**SW16**	**sk1**	**c2**
AbiEi/Eii_90730	$4 \times 10^{-2} \pm 8 \times 10^{-2}$	0.54 ± 0.07	0.27 ± 0.10	$6.6 \times 10^{-3} \pm 9.8 \times 10^{-3}$	0.61 ± 0.05	1.15 ± 0.34
AbiEi/Eii + up/downstream sequence_90730	0.42 ± 0.11	0.9 ± 0.03	0.95 ± 0.21	0.52 ± 0.10	1.01 ± 0.12	0.91 ± 0.06
Kiwa _90730	0.39 ± 0.02	0.67 ± 0.1	0.73 ± 0.17	0.34 ± 0.04	$5.17 \times 10^{-5} \pm 5.33 \times 10^{-6}$	$3.56 \times 10^{-4} \pm 5.68 \times 10^{-5}$
GAO_19 _R1	0.37 ± 0.07	0.86 ± 0.08	$0.26 \pm 6.9 \times 10^{-2}$	$3.8 \times 10^{-3} \pm 2.3 \times 10^{-3}$	1.52 ± 0.25	0.97 ± 0.06
Hachiman type I _ST19	1.33 ± 0.13	0.66 ± 0.06	0.35 ± 0.1	$8.4 \times 10^{-3} \pm 5 \times 10^{-3}$	1.18 ± 0.03	1.02 ± 0.05
AbiD_ST55	ND	0.59 ± 0.12	ND	ND	1.16 ± 0.26	0.88 ± 0.08
AbiD + up/downstream sequence_ST55	1.12 ± 0.07	0.46 ± 0.1	1.63 ± 0.35	$1.1 \times 10^{-2} \pm 6.0 \times 10^{-3}$	1.13 ± 0.03	1.14 ± 0.06
GAO_19 _ST4021	$\leq 1 \times 10^{-7}$	0.21 ± 0.05	0.50 ± 0.30	$4.0 \times 10^{-2} \pm 4.7 \times 10^{-3}$	$2.77 \times 10^{-5} \pm 7.16 \times 10^{-6}$	0.71 ± 0.11
AbiD_ST4021	0.23 ± 0.04	0.73 ± 0.25	$7.2 \times 10^{-2} \pm 2.7 \times 10^{-3}$	0.19 ± 0.03	1.07 ± 0.03	1.04 ± 0.10
AbiD + up/downstream sequence_ST4021	0.54 ± 0.11	0.56 ± 0.3	$0.29 \pm 8.2 \times 10^{-2}$	$3.0 \times 10^{-3} \pm 5 \times 10^{-3}$	1.03 ± 0.08	1.15 ± 0.32
AbiD_ST4078	0.44 ± 0.05	0.20 ± 0.07	0.20 ± 0.07	$1.52 \times 10^{-2} \pm 2.4 \times 10^{-3}$	$4.97 \times 10^{-4} \pm 1.5 \times 10^{-5}$	0.66 ± 0.19
AbiD + up/downstream sequence_ST4078	ND	ND	ND	ND	0.30 ± 0.04	0.72 ± 0.19
Gabija_ST4145	1.03 ± 0	0.46 ± 0.1	0.79 ± 0.11	0.34 ± 0.07	0.90 ± 0.06	0.90 ± 0.13
PD-T4-6_ST4078	ND	ND	ND	ND	0.96 ± 0.22	0.75 ± 0.08
PD-T4-6 + upstream sequence_ST4078	ND	ND	ND	ND	0.24 ± 0.03	0.48 ± 0.22
Dodola_90729	ND	0.7 ± 0.23	ND	ND	0.90 ± 0.10	0.67 ± 0.16
SoFic_MM20	1.41 ± 0.03	0.76 ± 0.06	0.71 ± 0.17	$5.8 \times 10^{-3} \pm 2.3 \times 10^{-3}$	0.80 ± 0.02	0.63 ± 0.03
